# Physiological characterization of electrodermal activity enables scalable near real-time autonomic nervous system activation inference

**DOI:** 10.1371/journal.pcbi.1010275

**Published:** 2022-07-28

**Authors:** Rafiul Amin, Rose T. Faghih

**Affiliations:** 1 Department of Electrical and Computer Engineering, University of Houston, Houston, Texas, United States of America; 2 Department of Biomedical Engineering, New York University, New York City, New York, United States of America; Inria, FRANCE

## Abstract

Electrodermal activities (EDA) are any electrical phxenomena observed on the skin. Skin conductance (SC), a measure of EDA, shows fluctuations due to autonomic nervous system (ANS) activation induced sweat secretion. Since it can capture psychophysiological information, there is a significant rise in the research work for tracking mental and physiological health with EDA. However, the current state-of-the-art lacks a physiologically motivated approach for real-time inference of ANS activation from EDA. Therefore, firstly, we propose a comprehensive model for the SC dynamics. The proposed model is a 3D state-space representation of the direct secretion of sweat via pore opening and diffusion followed by corresponding evaporation and reabsorption. As the input to the model, we consider a sparse signal representing the ANS activation that causes the sweat glands to produce sweat. Secondly, we derive a scalable fixed-interval smoother-based sparse recovery approach utilizing the proposed comprehensive model to infer the ANS activation enabling edge computation. We incorporate a generalized-cross-validation to tune the sparsity level. Finally, we propose an Expectation-Maximization based deconvolution approach for learning the model parameters during the ANS activation inference. For evaluation, we utilize a dataset with 26 participants, and the results show that our comprehensive state-space model can successfully describe the SC variations with high scalability, showing the feasibility of real-time applications. Results validate that our physiology-motivated state-space model can comprehensively explain the EDA and outperforms all previous approaches. Our findings introduce a whole new perspective and have a broader impact on the standard practices of EDA analysis.

## Author summary

The current state-of-the-art lacks physiology-motivated models for electrodermal activities (EDA) that have the power to comprehensively describe the variations in skin conductance (SC)–a measure of EDA. In this study, we propose a physiology-motivated state-space model to address previous challenges. On the other hand, there is also an absence of a scalable autonomic nervous system (ANS) activation inference method that simultaneously solve for the physiological system parameters. Furthermore, we develop a scalable ANS activation inference approach based on the proposed model with a goal for real-time edge computation. We utilize a dataset with 26 participants to validate the new model and the scalable method. Results demonstrate that our physiology-motivated state-space model can comprehensively explain the EDA. Our findings introduce a whole new perspective and have a broader impact on standard practices of EDA analysis.

## Introduction

The term “electrodermal activity” (EDA) refers to any electrical phenomenon on human skin [1]. EDA was discovered in the late 19th century and, since then, it has been widely used in psychophysiology as the EDA fluctuations have high correlations with the autonomic nervous system (ANS) activation. One of the most popular measures of EDA is the continuous exosomatic recording of skin conductance (SC). Due to emotional stimuli, there is a change in the psychophysiological and metabolic state of the body in order to deal with the emotional stimuli (e.g. flight or fight response). ANS may excite sweat glands based on the psychophysiological and metabolic change in the state, and the corresponding salty sweat secretions increase SC. Examination of SC measurements enables us to investigate ANS activation related to emotional arousal [2].

There are a few vital signals in the human body similar to EDA that have the potential to be measured continuously and unobtrusively using very simple instrumentation. The unobtrusive nature of the measuring techniques has led to a new era of wearable technology for continuous health monitoring. Such signals include cardiac signals (e.g. electrocardiogram (ECG) and photoplethysmogram (PPG)), skin temperature (SKT), EDA, muscle activity (e.g. electromyogram (EMG)) etc. [3, 4]. Among them, PPG and SKT have been widely integrated into consumer wearable technologies, along with reliable techniques for decoding useful information. In the past few decades, extensive research has been conducted, mainly on PPG signal analysis for wearable implementation, with the goal of continuous health monitoring. The next candidate with the greatest potential for revolutionizing wearable health monitoring is EDA [5]. However, the amount of research performed on EDA signals is relatively limited compared to cardiac signals. Although researchers have published many studies to systematically model EDA in the last two decades, there are still many fundamental characteristics of EDA being discovered today. For example, in 2020, Subramaniam *et al*. [6] have shown that the point process characterizes EDA in normal healthy participants. Therefore, further studies are required to identify the more accurate system dynamics of EDA so that critical information related to health monitoring can be obtained.

Appropriate EDA analysis has applications in a wide range of fields such as mental disorders, pain, cognitive stress tracking, wakefulness, etc. As different physiological signals, including EDA, contain information about human emotional arousal, they have potential applications in the field of mental health. For example, preventing death from mental disorders with regular tracking could be one potential application, as Walker *et al*. [7] reported that a large portion of deaths worldwide are attributable to mental health-related disorders. A meta-analysis shows that mental disorders are a major risk factor for suicide [8]. Suicide is one of the leading causes of death in the United States in the year 2017 [9] and the cost related to suicide alone in the United States were more than $90 billion in 2013 [10]. Studies have recommended [10] community-based immediate psychiatric services, including telepsychiatric support for reducing suicide-related costs which require continuous monitoring. Augmenting EDA with other physiological signals for time-to-time monitoring of critical patterns of emotional regulation could potentially help preventing psychiatric disorders [11].

Another possible potential application is in treating diabetic neuropathy. Diabetic neuropathy refers to small nerve damage caused by prolonged exposure to high levels of blood glucose concentration [12]. As a result, small nerves along with the sudomotor nerves in the legs, feet, and hands that are responsible for transmitting ANS activation are prone to neuropathy [12]. As confirmed by numerous studies in [13–15], damages in small nerves, including the sudomotor nerves may lead to abnormal EDA variations. Furthermore, it is well known in clinical diagnostics that the development of anomalies in sweat secretions may be attributed to forms of disorders, such as hypohidrosis and anhidrosis [16]. Moreover, such disorders may indicate diseases like diabetes mellitus [16]. Clinical investigations of abnormalities in the SC recordings can be pivotal for the early detection of such diseases.

Because of its wide range of applicability, accurate modeling of system-theoretic understanding is a prerequisite. In 1997, Lim et al. [17] proposed a heuristic sigmoid-exponential model to represent the rise and decay characteristics of the SCR shape. Instead of a general approach, they had to consider four different configurations of the proposed model for four different cases. Later in 2005, Alexander et al. [18] proposed a second-order differential equation for defining the SC fluctuations, the solution of which is a bi-exponential function representing the rise and decay of the SCR shape. They assumed that SC is single-phasic and, more specifically, that all fluctuations can be defined with the second-order differential equation. However, eventually researchers have realized the bi-phasic nature of EDA fluctuations, meaning there are two different components in EDA that vary in two different rates [19–24]. Bach *et al*. [25] have used a low-pass filter to separate slow varying component and then investigated the fast varying component as the output of a finite linear time-invariant (LTI) filter. Benedek *et al*. [19, 26] have suggested bi-exponential functions, namely Bateman functions, to describe the slow varying components with large decay time and the fast varying component with smaller decay times. However, this model cannot explain both components together. In a similar time, Bach *et al*. [20] reported that bi-exponential functions provided better fit than other candidates while modeling the fast varying component after removing the slow varying component with low-pass filter. Nevertheless, the FIR filter-based separation of the slow and fast varying components has limitations as pointed out in our previous work [24].

In our previous studies [23, 24, 27–29], we have developed deconvolution approaches in which we investigated previously known mathematical models for EDA dynamics. In these studies, we have utilized the SC modeling approach in [21], where the authors have modeled the slow varying component of EDA with a linear combination of a few arbitrary cubic spline basis functions. Although such a model can provide a good fit to the data, it lacks a reasonable physiological justification, and the corresponding coefficients of the obtained cubic-spline functions obtained do not have an interpretation. Furthermore, the cubic-spline basis function based model may overfit to the data and provide a solution that is not physiologically plausible. In addition, the lack of a complete state-space model makes it difficult to design scalable fixed-interval smoother (FIS) based inference approaches for recovery of ANS activation. Although similar approaches have been developed for calcium oscillation deconvolution and EEG sleep spindle detection [30], it is difficult to develop such an approach for EDA with the models currently available. During our development of deconvolution approaches, we realized that there is a need for a potential improvement in the current mathematical models for describing EDA dynamics as well as the current deconvolution practices to obtain a systematic and reliable approach with the feasibility of real-time application.

Therefore, in this study, we propose a unified and comprehensive state-space model to describe both the slow and fast varying components of EDA. We first start with a more general and physiologically interpretable nonlinear model and then derive a simpler linear state-space one. Additionally, our proposed model enables us to derive an FIS based novel scalable sparse deconvolution approach which was not previously possible because of the absence of a comprehensive state-space model for the potential of real-time inference. For obtaining our novel approach, we extended the scalable sparse deconvolution approach for calcium and EEG sleep spindle deconvolution proposed by Kazemipour et al. [30], which was developed for a subset of state-space equations considering the input matrix as an identity matrix. We generalized this for the state-space models with any input matrix and apply it for our proposed SC model. Moreover, for estimating the state-space model parameters, we utilize the previously known physiological priors similar to [24]. Furthermore, we employ generalized-cross-validation for balancing between the sparsity level of the ANS activation and the model fit for systematic reduction of the measurement noise. We compare the performance of our approach with previous deconvolution approaches. Furthermore, we show the scalability of our approach, illustrating the feasibility of devising real-time edge computation with our approach.

## Materials and methods

### Dataset description

In this study, we analyze the SC recordings where participants experience multiple auditory stimuli (loud sounds) during the experiment [31]. The experiment was designed to investigate event-related SC responses (SCRs) [32]. Each participants received multiple auditory stimuli. Each auditory stimulus is a single white noise burst of 1s length with a 10 ms ramp and 85 dB power. The participants were instructed to press a foot pedal upon hearing a stimulus. The dataset contains recordings from thirteen female and thirteen male participants. The partcipants are all healthy and unmedicated with age 24.4+/-4.9 years. For each of the 26 participants, the datasets include three channels of SC recordings from three different locations. We use the SC recordings from the thenar/hypothenar of the nondominant hand for all datasets in this study. The details regarding the experiment are provided in [32]. We pre-process all recordings with an approach similar to [28] and resample the SC recordings to 4 Hz for our analysis.

### Proposed physiological model

**Fig 1:**
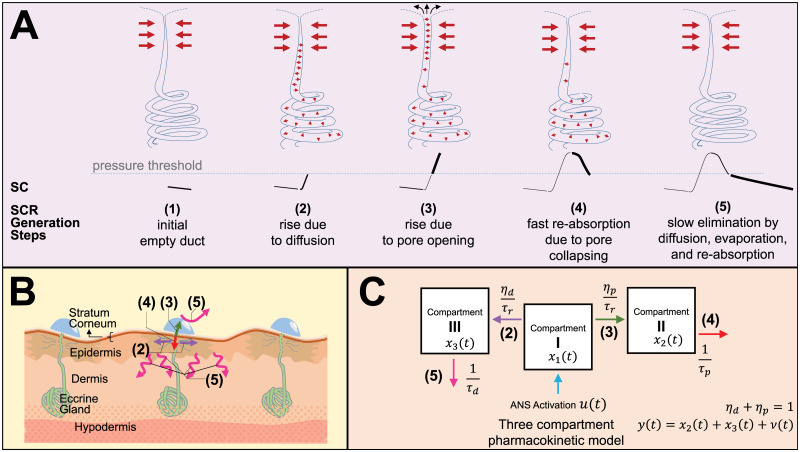
An overview of the physiology and corresponding proposed model. (A) A step by step illustration of the *poral valve model* proposed by Edelberg [33]. (B) An illustration of the cross section of the skin segment and corresponding different regions contributing to the SCR generation process based on *poral valve model*. (C) A three compartment pharmacokinetic realization of the *poral valve model*. The arrows with different colors in panel B and C correspond to the secretion and clearance of sweat contents in different steps denoted by the associated step numbers as represented in panel A.

We propose our model based on the *poral valve model* by Edelberg [33]. For the sake of discussion, let’s assume the sweat ducts are initially empty and in response to the received impulsive ANS activation, secretions from the sweat glands start to fill the sweat ducts. As the amount of sweat in the ducts increases, there is an increase in the hydraulic pressure inside. The pressure build-up gives rise to the increasing diffusion into the stratum corneum and the deeper stratum corneum area. This results in a slight rise in the SC level. If the pressure exceeds a certain threshold, the pores of the sweat ducts open for sweat secretion. This way, a fraction of the sweat is secreted directly by the pore opening. The secreted sweat and the connected sweat content in the ducts both contributes to the conductance. Therefore, there is a sharp rise in the SC level. Here, direct secretion refer to the secretion of sweat via the pore to the surface of the skin. On the other hand, sweat secretion via diffusion refers to hydration of stratum corneum when sweat slowly travels via the sweat duct wall. As the direct secretion and the diffusion reduces the hydraulic pressure and the pressure goes below a certain threshold, the pore collapse separates the sweat contents in the ducts and prevents them from contributing to the conductance. Consequently, a faster decay in SC level is observed. We define it as the faster re-absorption resulting in the faster decay time in SC. The remaining secreted fraction of the sweat in the stratum corneum is diffused into the deeper dermis and cleared away from the periductal area by a slow re-absorption process. Along with re-absorption, a fraction in the reduction of SC is because of the evaporation from the surface. These steps will lead to SC level to decay slowly. A visual illustration of the steps for the poral valve model is provided in Fig 1A. Fig 1B shows a cross section of the skin illustrating regions involved in different steps of SCR generation. With these speculations, we propose the following nonlinear state-space model:

$$\begin{array}{ccc} {\dot{x}}_{1}\left(t\right) & =-\frac{1}{\tau_{r}}x_{1}\left(t\right)+u\left(t\right), & \left(\text{sweat}\;\text{production}\right) \end{array}$$ (1)

$$\begin{array}{ccc} {\dot{x}}_{2}\left(t\right) & =\frac{\eta_{p}\left(x_{1}\left(t\right)\right)}{\tau_{r}}x_{1}\left(t\right)-\frac{1}{\tau_{p}}x_{2}\left(t\right), & \left(\text{pore}\;\text{opening}\;\text{and}\;\text{collapse}\right) \end{array}$$ (2)

$$\begin{array}{ccc} {\dot{x}}_{3}\left(t\right) & =\frac{\eta_{d}\left(x_{1}\left(t\right)\right)}{\tau_{r}}x_{1}\left(t\right)-\frac{1}{\tau_{d}}x_{3}\left(t\right) & \left(\text{slow}\;\text{re-absorption}\right) \end{array}$$ (3)

where *x*_1_(*t*), *x*_2_(*t*), and *x*_3_(*t*) respectively denote the states corresponding to the amount of sweat in the sweat ducts, in the ducts but electrically conducted to the surface due to the pore opening (contributing to the SC level), and diffused in the stratum corneum according to the hypothesis in the poral valve model proposed by Edelberg [33]. The states *x*_2_(*t*) and *x*_3_(*t*) are contributing to the rise in the SC level. *τ*_*p*_ denotes the faster decay time due to fast re-absorption (related to the pore collapse). *τ*_*d*_ represents the slow decay time related to the elimination from stratum corneum partially by re-absorption, diffusion in the deeper stratum corneum, and evaporation. We assume clearance rate from the sweat duct is equal to the sweat secretion rate to the surface and the adjacent skin tissue are. *τ*_*r*_ denotes the rise time of SC, (effectively the clearance time of the sweat from the ducts). One should note that the state-space model does not assume the duct is initially empty. Here, Eq 1 denotes the mechanism of ANS activation to the Compartment I for sweat production and corresponding sweat transportation towards Compartment II and III in Fig 1C. Eq 2 denotes the increase in the sweat content in Compartment II and corresponding fast re-absorption process in the model in Fig 1C. The location and the direction of the direct sweat secretion via pore opening (SCR generation step 3 in green arrow) and the corresponding fast re-absorption (SCR generation step 4 in red arrow) are denoted in Fig 1B and 1C. Similarly, Eq 3 denotes the increase in the sweat content in Compartment III and corresponding slow elimination process in the model in Fig 1C. The location and the direction of the sweat secretion via diffusion (SCR generation step 2 in purple arrow) and the corresponding fast re-absorption (SCR generation step 5 in magenta arrow) are denoted in Fig 1B and 1C.

The system input *u*(*t*) represents the ANS activation. To keep the definition simple, we assume that the ANS activation occurs during the integer multiple of the sampling period. Let *T*_*s*_ be the sampling period. Researchers reported that a single neural impulse from ANS is responsible for a single SC response [21, 22, 34–36]. Moreover, the sparsity constraint on *u* has been proven to be an appropriate prior in our previously developed algorithms [23, 24, 27–29, 37]. With the sparsity assumption, we represent the ANS activation as $u\left(t\right)=\sum_{k=1}^{K}u_{k}\delta \left(t-kT_{s}\right)$ where *u*_*k*_ is the amplitude of the impulse during the ANS activation at time *kT*_*s*_. *u*_*k*_ is zero if there is no impulse in the stimuli. Moreover, *η*_*p*_(*x*_1_(*t*)) and *η*_*d*_(*x*_1_(*t*)) are two functions that determine the fraction of sweat that is secreted by direct pore opening and diffusion, respectively. We assume *η*_*p*_(*x*_1_(*t*)) and *η*_*p*_(*x*_1_(*t*)) denote the nonlinearity in the pore opening operation. The nonlinearity of the pore opening is similar to the switching operation (on/off) and analogous to how a neuron works, i.e., in integrate-and-fire manner as pointed out in [6]. Therefore, we propose to model these nonlinearities with sigmoid functions similar to the artificial neurons as follows:

$$\begin{array}{c} \eta_{p}\left(x_{1}\left(t\right)\right)=S\left(\alpha x_{1}\left(t\right)+\beta \right),\\ \eta_{d}\left(x_{1}\left(t\right)\right)=1-S\left(\alpha x_{1}\left(t\right)+\beta \right) \end{array}$$

where *S*(*x*) = (1 + *e*^−*x*^)^−1^ represents the sigmoid function. Although we assume it as an integrate-and-fire operation, there is a difference, i.e., even if the pores do not open, the sweat secretion will still be carried out by the diffusion process via duct wall with relatively slower. Here, the nonlinear function *η*_*d*_(*x*_1_(*t*)) represents the the fraction of sweat secreted via diffusion for a given duct pressure represented by *x*_1_(*t*). Similarly, *η*_*p*_(*x*_1_(*t*)) represents the change in the fraction of sweat secreted via pore opening for a given duct pressure represented by *x*_1_(*t*). We assume thatthe amount of absorbed sweat in the stratum corneum and epidermis that contribute to the SC level due to diffusion process is denoted by *x*_3_(*t*). The sweat content in the ducts and electrically conducted to the surface due to the pore opening is denoted by *x*_2_(*t*) contribute to the SC level. Therefore, the observation equation denoting resultant SC is as follows,

$$y\left(t\right)=x_{2}\left(t\right)+x_{3}\left(t\right)+\nu \left(t\right)$$

where *y*(*t*) and *ν*(*t*) represent overall SC measurement and the noise signal, respectively. Equivalent to previous approaches, the phasic and the tonic components can be written as follows,

$$\begin{array}{c} y_{P}\left(t\right)=x_{2}\left(t\right)\\ \text{and}\;y_{T}\left(t\right)=x_{3}\left(t\right) \end{array}$$

where *y*_*P*_(*t*) and *y*_*T*_(*t*) represents the phasic and the tonic components, respectively.

Apparently, the proposed model is highly nonlinear and it is very difficult to derive a practical deconvolution approach that runs in edge devices with this model. For the simplification, we assume that the fraction of sweat secretion that happens via pore opening is always constant. Therefore, the simplified linear version of the model is obtained by the assumption that *η*_*p*_ and *η*_*d*_ is constant w.r.t *x*_1_(*t*) (*α* = 0) s.t. *η*_*d*_ = 1 − *η*_*p*_ = *η*. Here, *η* is a constant and it represents the fraction of sweat that is secreted by diffusion process, i.e., *η* ∈ [0, 1]. This simplification makes the model linear and more suitable for scalable edge computation. Now, the simplified model can be thought of as a three compartment pharmacokinetic model as shown in Fig 1C. To represent it in vector matrix form we define $x\left(t\right)={\left[\begin{array}{ccc} x_{1}\left(t\right) & x_{2}\left(t\right) & x_{3}\left(t\right) \end{array}\right]}^{⊤}$, $A_{c}=[\begin{array}{ccc} -\frac{1}{\tau_{r}} & 0 & 0\\ +\frac{\eta_{p}}{\tau_{r}} & -\frac{1}{\tau_{p}} & 0\\ +\frac{\eta_{d}}{\tau_{r}} & 0 & -\frac{1}{\tau_{d}} \end{array}]$, $B_{c}=[\begin{array}{c} 1\\ 0\\ 0 \end{array}]$, $C_{c}=[\begin{array}{ccc} 0 & 1 & 1 \end{array}]$. Therefore, the continuous state-space model in matrix form is as follows:

$$\begin{array}{c} \dot{x}\left(t\right)=A_{c}x\left(t\right)+B_{c}u\left(t\right),\\ y\left(t\right)=C_{c}x\left(t\right)+\nu \left(t\right). \end{array}$$

#### Discretization

Let *y*_*k*_ be the observed SC at time instance *kT*_*s*_. We can write,

$$\begin{array}{c} y_{k}=C_{c}y\left(kT_{s}\right)+\nu_{k} \end{array}$$ (4)

where *ν*_*k*_ ∀ *k* represent the noise and are modelled as independent and identically distributed (i.i.d) zero mean Gaussian random variable, i.e., $\nu_{k}∼N\left(0,\sigma_{\nu }^{2}\right)$. We derive the discrete equivalent of the system, assuming that the input and the states are constant over *T*_*s*_. The discrete version of the neural stimuli can be written as a vector ***u*** = [*u*_1_ *u*_2_ ⋯ *u*_*K*_]^⊤^ that represents the entire neural stimuli over the duration of SC data. Let $A=e^{A_{c}T_{s}}$, $B=\int_{0}^{T_{s}}e^{A_{c}\left(T_{s}-\rho \right)}B_{c}d\rho$, and ***C*** = ***C***_*c*_ to write the discrete state-space form as:

$$\begin{array}{c} x_{k}=Ax_{k-1}+Bu_{k},\;y_{k}=Cx_{k}+\nu_{k}. \end{array}$$ (5)

where $x_{k}\in R^{3}$, $y_{k}\in R$, *u*_*k*_, *ν*_*k*_ denote the state vector, the observation, ANS activation, and the measurement error in discrete domain. The corresponding discretized phasic and tonic components can be written as follows,

$$\begin{array}{c} y_{P,k}=C_{P}x_{k}\\ \text{and}\;y_{T,k}=C_{T}x_{k} \end{array}$$

where $C_{p}=[\begin{array}{ccc} 0 & 1 & 0 \end{array}]$ and $C_{T}=[\begin{array}{ccc} 0 & 0 & 1 \end{array}]$. Here, *y*_*P*,*k*_ and *y*_*T*,*k*_ represents the discretized version of the phasic and the tonic components, respectively.

### Physiological priors and constraints

The proposed model has many unknown parameters, and the number of measurements is relatively small. Therefore, the problem has many degrees of freedom. It is customary to enforce appropriate physiologically motivated priors on the model parameters. Otherwise, in the worst cases scenarios, the solution may not stay within the physiological boundaries and may lead to over-fitting [38]. Therefore, we incorporated physiologically motivated priors on the system model similar to [24, 39]. We assume that the individual model parameters are Gaussian distributed with some mean and variance similar to [24]. We use this information as a prior in the estimation step.

Further, we also consider equality and inequality constraints on the system parameters. First of all, we constraint all the physiological parameters are non-negative. We select a lower bound for *τ*_*r*_ of 0.2 seconds based on the result distribution obtained in our previous study [24]. Furthermore, we set *τ*_*p*_ > *β*_1_*τ*_*r*_ and *τ*_*d*_ > *β*_2_*τ*_*p*_ similar to our previous work [23, 24, 28]. However, the values of *β*_1_ and *β*_2_ are unknown for the proposed model. Therefore, we select the values of *β*_1_ and *β*_2_ by manually by investigating the results by trials and errors such that the multiple correlation coefficients for all participants are *R*^2^ > 0.98. First, we try to run the algorithm (described in the next section) without any constraint on *τ*_*r*_, *τ*_*p*_, *τ*_*d*_ and *η*. However, most of the case algorithm converges in a solution where the model fit is very poor and has a very small multiple correlation coefficient. And in most cases, *η* was convergent to 0 or 1. This is an indication of having a model with a very high degree of freedom. Therefore, we first decided to fix *η* = 0.5 assuming that 50% contributions of each type of secretion (i.e., via pore opening and via diffusion) reduce the complexity. Second, we decide to set as *τ*_*p*_ > 2*τ*_*r*_ as this constraint can be inferred from the previous distribution of the rise time and the decay time of the phasic component [24]. The reader should note that the estimated phasic decay time is at least 3 to 4 times the estimated rise time in [24]. Therefore, *β*_1_ = 2 should be a fairly conservative choice. Finally, we decide to find the constraint for *τ*_*d*_. As among all the time constants, *τ*_*d*_ is the slowest one, we consider the constraint with *τ*_*d*_ > *β*_2_*τ*_*p*_ for different *β*_2_ ≥ 1 and run the algorithm and try to see which value provide better goodness of fit for all 26 participants in terms of *R*^2^. We start with *β*_2_ = 1 and increment it by 1. We stop once all the participants (except Male Participant 12 as there is no fluctuation) show above 0.98 of *R*^2^. One should note that other configurations might also work. For example, if someone decides to start with a value of *η* other than 0.5, they might have to follow a similar procedure to find the new constraints. This suggests that there is a scope of further future investigation of the current method.

### Estimation

We wish to estimate the parameter vector $\theta ={\left[\begin{array}{ccccc} \theta_{1} & \theta_{2} & \theta_{3} & \theta_{4} & \theta_{5} \end{array}\right]}^{⊤}={\left[\begin{array}{ccccc} \tau_{r} & \tau_{p} & \tau_{d} & \eta_{p} & \eta_{d} \end{array}\right]}^{⊤}$ and unknown ANS activation *u*_*k*_ given the SC measurement *y*_*k*_ ∀ *k* ∈ {0, 1, ⋯, *K* − 1}. One straighforward way is to solve the following optimization problem,

$$\begin{array}{cc} & \underset{x_{k},\;\forall k,\;\theta_{j},\;\forall J}{\text{min}}\;\lambda \sum_{k=0}^{K-1}\left|\right|x_{k}-Ax_{k-1}{\left|\right|}_{1}+\sum_{k=0}^{K-1}\frac{\left|\right|y_{k}-Cx_{k}{\left|\right|}_{2}^{2}}{2\sigma_{\nu }^{2}}\\ & \;\;\;\;\;\;\;\;\;\;\;\;\;\;\;+\sum_{j=0}^{j=J-1}\rho_{j}\frac{{\left(\theta_{j}-{\bar{\theta }}_{j}\right)}^{2}}{2\sigma_{\theta_{j}}^{2}}. \end{array}$$ (6)

where (***x***_*k*_ − *A****x***_*k*−1_) = ***B****u*_*k*_. If we consider the first term in Eq 6, i.e., the *l*_1_-norm of (***x***_*k*_ − *A****x***_*k*−1_) as the negative log-likelihood, taking the exponential of the negative of the gives us the Laplace distribution of ***B****u*_*k*_ = (***x***_*k*_ − *A* ***x***_*k*−1_) with parameter λI. The second term in Eq 6 represents the least squares error between the observation *y*_*k*_ and the prediction *C****x***_*k*_ with a Gaussian observation error assumption. The final term represents the negative loglikelihood of the Gaussian priors on the system parameters with *ρ*_*j*_, ${\bar{\theta }}_{j}$, and $\sigma_{\theta_{j}}$ represents the regularization parameters, the mean, and variance for the Gaussian priors, respectively ∀*j* ∈ {0, 1, 2, ⋯, *J* − 1}. In this case, *J* = 3. Therefore, Eq 6 can be considered as the maximum *a posterior* (MAP) estimator as pointed out in [30]. In general, the problem formulation in Eq 6 is solved for *u*_*k*_ by taking the derivative of Eq 6 with respect *u*_*k*_ and setting it zero. This is particularly done using iteratively re-weighted least square (IRLS) approach. The sparse recovery with the direct analytical solution of the state-space model requires a matrix inversion of a *K* × *K* matrix as shown in our previous works [23, 24, 27]. This step works as the bottle neck of the approach. In this study, we solve the very same problem with iterative re-weighted lease squares approach implemented using FIS. The states ***x***_*k*_, the ANS activation *u*_*k*_ and the matrices describing system dynamics ***A*** and ***B*** can be estimated in an expectation-maximization (EM) approach.

Given the probabilistic model that generates a set of observed data *Y* = {*y*_*k*_}∀*k* ∈ {0, 1, ⋯, *K* − 1} and a vector of unknown parameters ***θ***, we can write, *p*(*Y*, *θ*) = *p*(*Y*|*θ*)*p*(*θ*). The following maximum log-likelihood estimation problem can be solved in order to estimate the *θ*:

$$\underset{\theta }{\text{max}}\;\text{log}\;p(Y;\theta )$$

Now lets introduce a set of hidden unknown states *X* = {***x***_*k*_, *u*_*k*_} ∀ *k* having a joint probability distribution *p*(*Y*, *X*; *θ*). We can re-write the maximum likelihood estimation as the following marginal likelihood function of *p*(*Y*, *X*; *θ*):

$$\begin{array}{c} \underset{\theta }{\text{max}}\;\text{log}\;p(Y;\theta )=\underset{\theta }{\text{max}}\;\text{log}\int_{X}p\left(Y,X;\theta \right)dX. \end{array}$$ (7)

We defined the joint log-likelihood function for *Y*, *X*, and ***θ*** as follows:

$$\begin{array}{cc} & \text{log}\;p(Y,X;\theta )=\text{log}(p(Y|X,\theta )p(X|\theta )p(\theta ))\\ & =\text{log}\;p(Y|X,\theta )+\text{log}\;p(X|\theta )+\text{log}(\theta )\\ & =\sum_{k=0}^{K-1}\text{log}\left(p_{\nu_{k}}\left(y_{k}-Cx_{k}\right)\right)+\sum_{k=0}^{K-1}\text{log}\left(p_{Bu_{k}}\left(x_{k}-Ax_{k-1}\right)\right)\\ & \;\;\;\;\;\;\;\;\;\;\;\;\;\;\;+\text{log}(p(\theta )). \end{array}$$ (8)

where the $p_{\nu_{k}}$ and $p_{Bu_{k}}$ denotes the probability density functions corresponding to *ν*_*k*_ = *y*_*k*_ − ***Cx***_*k*_ and ***B****u*_*k*_ = ***x***_*k*_ − *A****x***_*k*−1_, respectively. Here, only the term $p_{Bu_{k}}\left(x_{k}-Ax_{k-1}\right)$ depends on *θ*.

The original problem can be defined as the following expectation maximization (EM) approach,

$$\begin{array}{c} \underset{\theta }{\text{max}}\;\text{log}\;p(Y;\theta )=\underset{\theta }{\text{max}}\;E_{X∼q(X)}\left\{\text{log}\;p(Y,X;\theta )\right\}. \end{array}$$ (9)

As it is expressed in Eq 9, the unknowns can be estimated by iteratively maximizing the expectation of the joint log-likelihood in Eq 8 as shown in S1 Appendix.

#### E-step (sparse recovery)

Let’s assume that we know the current estimate of model parameters ***θ***^(*i*−1)^ from the (*i* − 1)^th^ iteration of EM. We calculate the corresponding state matrices ***A***^(*i*−1)^ and ***B***^(*i*−1)^. At *i*^th^ iteration of EM, given the sequence of observations *y*_*k*_ ∈ *Y* and given probability distribution *q*(*X*) = *p*(*X*|*Y*, ***θ***^(*i*−1)^), we wish to estimate the expectation of $x_{k}^{\left(i\right)}$ and $u_{k}^{\left(i\right)}$. We choose the probability distribution for *u*_*k*_ such that it enforces sparsity. Kazemipour et al. [30] proposed to use Laplace distributed with parameter for sparsity of the innovation terms in the state transition equations. In this study, we consider a broader family of distributions, namely, generalized Gaussian distribution for *u*_*k*_ so that distribution parameters can be selected to obtain a range of distributions such as Gaussian and Laplace distribution. In contrast to [30] where the input matrix is considered as an identity one, we assume that $u_{k}^{\left(i\right)}$ denote the scalar (or column vector) ANS activation and ***B***^(*i*−1)^ works as a direction vector (or matrix) of innovation in the state transition equation. We consider $u_{k}^{\left(i\right)}$ is generalized Gaussian distributed, i.e.,

$$p\left(u_{k}^{\left(i\right)}|\gamma^{\left(i\right)},p\right)=\frac{p\gamma^{\left(i\right)}}{4\gamma^{\left(i\right)}\left(1/p\right)}\text{exp}(-\frac{\gamma^{\left(i\right)}}{2}|u_{k}^{\left(i\right)}{|}^{p}),$$

where *γ*^(*i*)^ and *p* defines the shape of the generalized Gaussian distribution. *p*(*u*_*k*_|*γ*^(*i*)^, *p*) can also be written in terms of ***x***_*k*_ with multi-variate generalized Gaussian distribution as follows.

$$\begin{array}{c} p\left(u_{k}^{\left(i\right)}|\gamma^{\left(i\right)},p\right)=p\left(Bu_{k}^{\left(i\right)}|\lambda^{\left(i\right)},p\right)=\text{exp}(-\frac{\lambda^{\left(i\right)}}{2}\left|\right|B^{\left(i-1\right)}u_{k}^{\left(i\right)}{\left|\right|}_{p}^{p})\\ =\text{exp}(-\frac{\lambda^{\left(i\right)}}{2}\left|\right|x_{k}^{\left(i\right)}-A^{\left(i-1\right)}x_{k-1}^{\left(i\right)}{\left|\right|}_{p}^{p}), \end{array}$$

where λ^(*i*)^ represents the new parameter related to the new random variable to obtain the equivalent pdf ($\lambda^{\left(i\right)}\left|\right|B^{\left(i-1\right)}{\left|\right|}_{p}^{p}=\gamma^{\left(i\right)}$). The sparsity constraint is imposed on $u_{k}^{\left(i\right)}$ for 0 < *p* < 2. However, the closed form equations for FIS do not exist for generalized Gaussian distribution where *p* ≠ 2, although they are the prerequisite for scalable edge computation of the sparse recovery. Therefore, we approximate the generalized Gaussian distribution with iterative re-weighted Gaussian distributions for the closed form derivation of the forward filter and backward smoother equations. For example, if *p* = 1, the generalized Gaussian distribution becomes Laplace distribution as shown in [30]. Therefore, we approximate the Laplace distribution of $u_{k}^{\left(i\right)}$ with iterative re-weighted Gaussian distributions, i.e., if at *r*^th^ re-weighting step the state estimation is $x_{k}^{\left(i,r\right)}$, the Laplace pdf can be approximated with Gaussian pdf as follows:

$$\begin{array}{c} p_{x_{k}}=\frac{\lambda^{\left(i,r\right)}}{2}\text{exp}(-\frac{\lambda^{\left(i,r\right)}}{2}{\left|\right|x_{k}^{\left(i,r\right)}-A^{\left(i-1\right)}x_{k-1}^{\left(i,r\right)}\left|\right|}_{1})\\ \propto \frac{\lambda^{\left(i,r\right)}}{2}\text{exp}(-\frac{1}{2}{\left(x_{k}^{\left(i,r\right)}-A^{\left(i-1\right)}x_{k-1}^{\left(i,r\right)}\right)}^{⊤}{\left(Q_{k}^{\left(i,r-1\right)}\right)}^{-1}\left(x_{k}^{\left(i,r\right)}-A^{\left(i-1\right)}x_{k-1}^{\left(i,r\right)}\right)), \end{array}$$

where λ^(*i*, *r*)^ is the regularization at *r*^th^ re-weighting step. $Q_{k}^{\left(i,r\right)}$ is the co-variance matrix at *r*^th^ re-weighting step at *k*^th^ time point and we define it defined as follows:

$$\begin{array}{cc} Q_{k}^{\left(i,r\right)} & ={\left(\lambda^{\left(i,r\right)}\right)}^{-1}{\left(E\left\{\left(x_{k}^{\left(i,r\right)}-A^{\left(i-1\right)}x_{k-1}^{\left(i,r\right)}\right){\left(x_{k}^{\left(i,r\right)}-A^{\left(i-1\right)}x_{k-1}^{\left(i,r\right)}\right)}^{⊤}\right\}+\epsilon^{2}I\right)}^{\frac{1}{2}}\\ & ={\left(\lambda^{\left(i,r\right)}\right)}^{-1}{\left(\left(B^{\left(i-1\right)}{\left(u_{k}^{\left(i,r\right)}\right)}^{2}{B^{\left(i-1\right)}}^{⊤}\right)+\epsilon^{2}I\right)}^{\frac{1}{2}}. \end{array}$$

Here, *ϵ* is a value close to zero for the matrix perturbation to achieve numerical stability. We select *ϵ* = 10^−5^ for the numerical stability. Unlike the conventional IRLS approach where the covariance of the Gaussian approximation is taken to be diagonal, here the current definition takes the square root of the entire matrix. The perturbations enable us to obtain feasible inverse during FIS prediction and update equations as $B^{\left(i-1\right)}{\left(u_{k}^{\left(i,r\right)}\right)}^{2}{\left(B^{\left(i-1\right)}\right)}^{⊤}$ is always singular. The generalized approximation is performed by implementing *ℓ*_*p*_-norm with Gaussian distribution approximation of generalized Gaussian family as follows where 0 < *p* < 2,

$$\begin{array}{cc} Q_{k}^{\left(i,r\right)} & ={\left(\lambda^{\left(i,r\right)}\right)}^{-1}{\left(\left(B^{\left(i-1\right)}{\left(u_{k}^{\left(i,r\right)}\right)}^{2}{\left(B^{\left(i-1\right)}\right)}^{⊤}\right)+\epsilon^{2}I\right)}^{\frac{2-p}{2}} \end{array}$$ (10)

Similar to the previous case with square root, here the power $\frac{2-p}{2}$ has been taken on the whole matrix. With this approximation, we perform Kalman filtering and backward smoothing to obtain the expectation of the state variables $E\{x_{k}^{\left(i,r\right)}\}$’s and corresponding covariance matrices. Constraining the corresponding innovation in the state equation to be along the direction of the vector ***B***, the expected *u*_*k*_ is given as follows at *r*^th^ re-weighting step:

$$\begin{array}{c} u_{k}^{\left(i,r\right)}=\underset{u\geq u_{\text{th}}}{\text{arg min}}\frac{1}{2}||E\left\{x_{k+1}^{\left(i,r\right)}\right\}-A^{\left(i-1\right)}E\left\{x_{k}^{\left(i,r\right)}\right\}-B^{\left(i-1\right)}u{\left|\right|}_{2}^{2}, \end{array}$$ (11)

where *u*_th_ is the selected minimum amplitude for ANS activation. Here, we select *u*_th_ = 0.03 *μS*/*s* for the initialization step and *u*_th_ = 0.25 *μS*/*s* during the main step. Here, a relatively conservative value of *u*_*th*_ has been selected in the initialization step to avoid excessive pruning before having good initialization of a other parameters. The selection has been done manually by trial and error such that the results for all participants that reduces the number of detected spikes while keeping the multiple correlation coefficient *R*^2^ > 0.95. During this process, the number of detected detected spikes that visually does not correspond to any SCR is minimized. The evaluation of the obtained spikes has been evaluated by visual inspection (verified by two different viewers) similar to apporach in [6]. The criteria of selecting *u*_th_ is chosen to obtain a reasonable goodness-of-fit define by *R*^2^ while avoiding any over-fitting. The use of threshold *u*_th_ enables us to obtain a constrained solution of *u*_*k*_ without implementing actual constrained Kalman filtering and backward smoothing. As $u_{k}^{\left(i,r\right)}$ is scalar in the above optimization formulation, the solution can be written directly as follows:

$$\begin{array}{c} u_{k}^{\left(i,r\right)}=\text{max}\left(u_{\text{th}},{\left({B^{\left(i-1\right)}}^{⊤}B^{\left(i-1\right)}\right)}^{-1}{\left(B^{\left(i-1\right)}\right)}^{⊤}\left(x_{k+1}^{\left(i,r\right)}-A^{\left(i-1\right)}x_{k}^{\left(i,r\right)}\right)\right), \end{array}$$ (12)

This allows us to project the error vector along the direction of ***B***^(*i*−1)^ vector based on least square error with a minimum threshold. This is an approximation to make sure that the solution is consistent with the assumptions of the state-space model. In this study, we select *p* = 0.5 for *l*_*p*_-norm similar to our previous studies in [23, 24, 27–29].

#### Adjust sparsity level by choosing *γ*

In the initialization phase, we choose a scheme for selecting λ similar to IRLS algorithm *FOCUSS*+ algorithm in [40]. At *r*^th^ re-weighting iteration of E-step, the heuristic estimation of λ works as follows:

$$\begin{array}{c} \gamma^{\left(i,r\right)}=(1-\sum_{k=0}^{K-1}\left|\right|y_{k}-Cx_{k}^{\left(i,r-1\right)}{\left|\right|}_{2}^{2}/\sum_{k=0}^{K-1}\left|\right|y_{k}{\left|\right|}_{2}^{2})\gamma^{\text{max}},\;\gamma >0 \end{array}$$ (13)

Then, we set $\lambda_{n}^{\left(i,r\right)}=\gamma_{n}^{\left(i,r\right)}/\left|\right|B^{\left(i-1\right)}{\left|\right|}_{p}^{p}$. Similarly, in the main EM phase, we use generalized-cross-validation (GCV) technique similar to the GCV-FOCUSS+ technique [41]. We modified the GCV technique to obtain scalability. To achieve this, we segment our observations with a window size of *M*_*gcv*_ samples and apply GCV to obtain a λ for each window. For *n*^th^ segment, the discretized vector form solution can be provided as, ${\tilde{y}}_{n}=F_{n}{\tilde{x}}_{n,0}+D_{n}{\tilde{u}}_{n},$ where ${\tilde{y}}_{n}$, ***x***_*n*+1_, ${\tilde{u}}_{n}$ represents the observation vector, the first state and the ANS activation in the *n*^th^ segment, respectively. ***F***_*n*_ and ***D***_*n*_ are the matrices for the complete discretized vector solution for *n*^th^ block and can be defined as, $F_{n}={\left[\begin{array}{cccc} F_{n,0} & F_{n,1} & \cdots & F_{n,(M_{gcv}-1)} \end{array}\right]}_{M_{gcv}\times 3}^{⊤}$ and $D_{\theta }={\left[\begin{array}{cccc} D_{n,0} & D_{n,1} & \cdots & D_{n,(M_{gcv}-1)} \end{array}\right]}_{M_{gcv}\times M_{gcv}}^{⊤},\;$ where ***F***_*n*,*k*_ = ***CA***^*k*^ and $D_{n,k}=C[\begin{array}{ccccc} A^{k-1}B & A^{k-2}B & \cdots & B & \underset{M_{gcv}-k}{\underset{︸}{0\;\cdots \;0}} \end{array}]$. *M*_*gcv*_ = 100 worked well for our study.

For *n*^th^ segment, we obtain λ_*n*_ using the following optimization formulation based on singular value decomposition (SVD) for GCV proposed in [41]:

$$\begin{array}{cc} & \underset{\lambda_{n}}{\text{min}}\;G_{n}\left(\gamma_{n}\right)=\frac{\left[M_{gcv}\sum_{n^{\prime }=1}^{M_{gcv}}{\hat{y}}_{n,n^{\prime }}^{2}{\left(\frac{\gamma_{n}}{\kappa_{n,n^{\prime }}^{2}+\gamma_{n}}\right)}^{2}\right]}{\left[\sum_{n^{\prime }=1}^{M_{gcv}}{\left(\frac{\gamma_{n}}{\kappa_{n,n^{\prime }}^{2}+\gamma_{n}}\right)}^{2}\right]}\\ & \text{s.t.}\;\;0\leq \gamma_{n}\leq 1\times 10^{-4} \end{array}$$ (14)

where $\hat{y}=U^{⊤}{\hat{y}}_{n,\tau }={\left[\begin{array}{cccc} {\hat{y}}_{n,1} & {\hat{y}}_{n,2} & \cdots & {\hat{y}}_{n,M_{gcv}} \end{array}\right]}^{⊤}$ with ${\hat{y}}_{n,\tau }={\tilde{y}}_{n}-F_{n}{\tilde{x}}_{n}$, and $D_{n}{P_{\tilde{u}}}^{\frac{1}{2}}=U\Sigma V^{⊤}$ with $P_{\tilde{u}}=\text{diag}\left(|{\tilde{u}}_{n,n^{\prime }}{|}^{2-p}\right)$ and **Σ** = diag{*κ*_*j*_}; ***U*** and ***V*** are unitary matrices and *κ*_*i*_’s are the singular values of $D_{n}P_{\tilde{u}}^{\frac{1}{2}}$. We estimate *γ*_*n*_∀*n* and take the median. Finally, we set $\lambda_{n}^{\left(i,r\right)}=\gamma_{n}^{\left(i,r\right)}/\left|\right|B^{\left(i-1\right)}{\left|\right|}_{p}^{p}$.

Usually, the re-weighting in E-step converges within a very small number of iterations. We perform the re-weighting in E-step for *r* = 0, 1, 2, ⋯, 5. After finishing all the re-weighting iterations in the E-step, we obtain the following estimations: $x_{k}^{\left(i\right)},u_{k}^{\left(i\right)},\;P_{k|k}^{\left(i\right)},$ and $\;P_{k|k-1}^{\left(i\right)}\forall k$. Here, $P_{k|k}^{\left(i\right)}$ and $P_{k|k-1}^{\left(i\right)}$ represents the estimates of $E\{x_{k}^{\left(i\right)}{x_{k}^{\left(i\right)}}^{⊤}\}$ and $E\{x_{k}^{\left(i\right)}{x_{k-1}^{\left(i\right)}}^{⊤}\}$, respectively. Here, we drop *r* to represent the final E-step estimations.

#### M-step (physiological parameter estimation)

The M-step at *i*^th^ iteration can be defined as the following simplified constrained optimization problem utilizing Eq 6 and EM derivation,

$$\begin{array}{cc} & \underset{\theta_{j},\;\forall j}{\text{min}}\;E\{\lambda^{\left(i\right)}\sum_{k=0}^{K-1}\left|\right|x_{k}^{\left(i\right)}-Ax_{k-1}^{\left(i\right)}{\left|\right|}_{p}^{p}+\sum_{k=0}^{K-1}\frac{\left|\right|y_{k}-Cx_{k}^{\left(i\right)}{\left|\right|}_{2}^{2}}{2\sigma_{\nu }^{2}}\\ & \;\;\;\;\;\;\;\;\;\;\;\;\;\;\;+\sum_{j=0}^{j=J}\rho_{j}\frac{{\left(\theta_{j}-{\bar{\theta }}_{j}\right)}^{2}}{2\sigma_{\theta_{j}}^{2}}\},\\ & \text{s.t.}\;R\theta \leq s,\;R_{e}\theta =s_{e}, \end{array}$$ (15)

where $R=[\begin{array}{ccccc} -1 & 0 & 0 & 0 & 0;\\ 0 & -1 & 0 & 0 & 0\\ 0 & 0 & -1 & 0 & 0\\ \beta_{1} & -1 & 0 & 0 & 0\\ 0 & \beta_{2} & -1 & 0 & 0 \end{array}]$, $s=[\begin{array}{c} s_{1}\\ s_{2}\\ s_{3}\\ 0\\ 0 \end{array}]$, $R_{e}=[\begin{array}{ccccc} 0 & 0 & 0 & 1 & 0\\ 0 & 0 & 0 & 0 & 1 \end{array}]$, and $s_{e}=[\begin{array}{c} 1-\eta \\ \eta \end{array}]$ determines the constraints on *θ*. The equality constraints ensures the sum of *η*_*p*_ and *η*_*d*_ are equal to 1. To incorporate estimated $u_{k}^{\left(i\right)}$ from the E-step, we re-write the Eq 15. The modified optimization formulation is as follows,

$$\begin{array}{cc} & \underset{\theta_{j},\;\forall j}{\text{min}}\;E\{\frac{\gamma^{\left(i\right)}}{2}\sum_{k=0}^{K-1}{\left|u_{k}^{\left(i\right)}\right|}^{p}+\sum_{k=0}^{K-1}\frac{\left|\right|y_{k}-C\left(Ax_{k-1}^{\left(i\right)}+Bu_{k}^{\left(i\right)}\right){\left|\right|}_{2}^{2}}{2\sigma_{\nu }^{2}}\\ & \;\;\;\;\;\;\;\;\;\;\;+\sum_{j=0}^{j=J}\rho_{j}\frac{{\left(\theta_{j}-{\bar{\theta }}_{j}\right)}^{2}}{2\sigma_{\theta_{j}}^{2}}\}\\ & \text{s.t.}\;R\theta \leq s,\;R_{e}\theta =s_{e} \end{array}$$ (16)

After some algebraic manipulation and assumption that $x_{k-1}^{\left(i\right)}$ and $u_{k}^{\left(i\right)}$ are statistically independent ∀*k*, we obtain the following optimization formulation by removing the constant terms with respect to ***θ***.

$$\begin{array}{cc} & \underset{\theta_{j},\;\forall j}{\text{min}}\;\;\frac{1}{2}{\left||y|\right|}^{2}+\frac{1}{2}Tr\left(A\left(\sum_{k=0}^{K-1}\left(x_{k-1}^{\left(i\right)}{\left(x_{k-1}^{\left(i\right)}\right)}^{⊤}+P_{k-1}^{\left(i\right)}\right)\right)A^{⊤}\right)\\ & \;\;\;-Tr\left(A\left(\sum_{k=0}^{K-1}y_{k}^{⊤}Cx_{k}^{\left(i\right)}\right)\right)-Tr\left(B\left(\sum_{k=0}^{K-1}y_{k}^{⊤}Cu_{k}^{\left(i\right)}\right)\right)\\ & \;\;\;+Tr\left(B\sum_{k=0}^{K-1}\left({\left(u_{k}^{\left(i\right)}\right)}^{2}\right)B^{⊤}\right)+Tr\left(Ax_{k-1}^{\left(i\right)}{\left(u_{k-1}^{\left(i\right)}\right)}^{⊤}B^{⊤}\right))\\ & \;\;\;+\sigma_{v}^{2}\sum_{j=0}^{j=J}\rho_{j}\frac{{\left(\theta_{j}-{\bar{\theta }}_{j}\right)}^{2}}{2\sigma_{\theta_{j}}^{2}}\\ & \text{s.t.}\;R\theta \leq s,\;R_{e}\theta =s_{e} \end{array}$$ (17)

The overall approach can be divided into two phases. In the first phase, we perform initialization with a fixed $u_{k}^{\left(i,0\right)}=u_{\alpha }\;\forall k$ at each iteration and with heuristic refinement of λ^(*i*, *r*)^. A detailed description of heuristic refinement is provided in S2 Appendix. *u*_*α*_ = 1 worked well for our study. In the main EM-phase, we update $u_{k}^{\left(i,0\right)}=u_{k}^{\left(i-1,5\right)}$, i.e. with the values obtained in the previous re-weighting iteration. In E-steps of both phases, we perform a heuristic refinement of *u*_*k*_. After finishing all re-weighting iterations in the E-step, we obtain the following estimations: $x_{k}^{\left(i\right)},u_{k}^{\left(i\right)},\;P_{k|k}^{\left(i\right)},$ and $\;P_{k|k-1}^{\left(i\right)}\forall k$. The expected values are plugged into the M-step optimization formulation in 17. The constrained optimization problem in 17 is solved using the interior-point method. The overall algorithm for the initialization and the main EM-phase is provided in Algorithm 1.

### Selection of noise variance *σ*_*ν*_ and it’s relation with λ

The presence of noise may lead to inaccurate estimates of ANS activations. The regularization parameter λ related to sparsity dictates the level sparsity of *u*_*k*_, choice of higher value of λ leads to more sparse solution and vice versa. On the other hand, if the of guess of the observation noise variance is higher, the estimation deconvolution tend to fit more to the state equation itself without having much innovation term (i.e. smaller ***B****u*_*k*_) than the current observation. For regular FIS, there is a always a trade-off between process noise and the observation noise. If the observation noise is high then the process noise usually tend be very low during the estimation. In case of IRLS-based FIS for sparse recovery, the process noise is represented with the innovation term, i.e. the ANS activation *u*_*k*_. Therefore, if the observation noise variance *σ*^2^ is selected to be smaller, the innovation *u*_*k*_ will have more zeros ∀*k*. In other words, higher value of observation noise variance *σ*^2^ leads to a more sparse estimation of *u*_*k*_. Therefore, although we have incorporated a GCV based approach for selecting λ that tunes the sparsity level of *u*_*k*_, the noise filtration also depends on the selected observation noise variance, $\sigma_{\nu }^{2}$. For the experimental study, we have selected $\sigma_{\nu }^{2}=1\times 10^{-8}$. This value is working well along with the GCV for balancing between discarding the noise and capturing the process. We have kept the value of $\sigma_{\nu }^{2}$ same for the simulated study. Our results show that it is capturing more spikes than the ground truth for heavy noise level. As pointed out in [30], increasing the noise variance $\sigma_{\nu }^{2}$ will lead to a much smoother estimate with a lower number of spikes. For most of the cases, GCV could discard most of the spikes related to noise. Because, the corresponding selected $\sigma_{\nu }^{2}$ are within the reasonable range for GCV to obtain a balance. Therefore, for GCV to balance the noise spike, a reasonable choice of $\sigma_{\nu }^{2}$ is required. However, for some cases it is challenging to find such a reasonable value for GCV. Higher values of $\sigma_{\nu }^{2}$ may result in some of the SCRs undetected. Therefore, we select a relatively small value of $\sigma_{\nu }^{2}$ such that none of the SCRs remain undetected. As most of the detected noise spikes are relatively smaller than the spikes related to the SCRs, application tailored post-processing (e.g. hard/soft thresholding) can remove most of the noise spikes.

**Algorithm 1**: bayesianEDA

**Input**: *y*_*k*_ ∀*k*

**Output**: *u*_*k*_ ∀*k* and *θ*

**1 Initialization Phase**: Initialize ${\tilde{\theta }}^{0}∼U\left(b_{l},b_{u}\right)$.

**2 for** *i* = 1, 2, 3, ⋯, 30 **do**

**3**  Set $u_{k}^{\left(i,0\right)}=u_{\alpha }\;\forall k$

**4**  **E-Step**:

**5**  With $\theta ={\tilde{\theta }}^{\left(i-1\right)}$, calculate ***A***^(*i*−1)^ and ***B***^(*i*−1)^

**6**  **Iterative re-weighting**:

**7**  **for** *r* = 1, 2, 3, ⋯, 10 **do**

**8**   Estimate λ^(*i*,*r*)^ using 13.

**9**   Perform heuristic refinement of $u_{k}^{\left(i,r-1\right)}$.

**10**   Set $Q_{k}^{\left(i,r-1\right)}={\left(\lambda^{\left(i,r\right)}\right)}^{-1}{\left(\left(B^{\left(i-1\right)}{\left(u_{k}^{\left(i,r-1\right)}\right)}^{2}{\left(B^{\left(i-1\right)}\right)}^{⊤}\right)+\epsilon^{2}I\right)}^{\frac{2-p}{2}}$.

**11**   Estimate $x_{k}^{\left(i,r\right)},\;P_{k|k}^{\left(i,r\right)}$ and $P_{k|k-1}^{\left(i,r\right)}$ using FIS.

**12**   Set $u_{k}^{\left(i,r\right)}=\text{max}\left(u_{\text{th}},{\left({B^{\left(i-1\right)}}^{⊤}B^{\left(i-1\right)}\right)}^{-1}{B^{\left(i-1\right)}}^{⊤}\left(x_{k}^{\left(i,r\right)}-Ax_{k-1}^{\left(i,r\right)}\right)\right)$.

**13**  **end**

**14**  **M-Step**: Set $x_{k}^{\left(i\right)}=x_{k}^{\left(i,r\right)},\;u_{k}^{\left(i\right)}=u_{k}^{\left(i,r\right)},\;P_{k|k}^{\left(i\right)}=P_{k|k}^{\left(i,r\right)}$ and $P_{k|k-1}^{\left(i\right)}={P_{k|k-1}}^{\left(i,r\right)}$ and solve the optimization problem in Eq 17 to obtain ***θ***^(*i*)^.

**15 end**

**16 Main EM Phase**: **while** until convergence **do**

**17**  Set *i* = *i* + 1

**18**  Set $u_{k}=u_{k}^{\left(i-1,r\right)}\;\forall k$

**19**  **E-Step**:

**20**  With $\theta ={\tilde{\theta }}^{\left(i-1\right)}$, calculate ***A***^(*i*−1)^ and ***B***^(*i*−1)^.

**21**  **Iterative re-weighting**:

**22**  **for** *r* = 1, 2, 3, ⋯, 10 **do**

**23**   Estimate λ^(*i*, *r*)^ using the modified GCV technique.

**24**   Perform heuristic refinement of $u_{k}^{\left(i,r-1\right)}$.

**25**   Set $Q_{k}^{\left(i,r-1\right)}={\left(\lambda^{\left(i,r\right)}\right)}^{-1}{\left(\left(B^{\left(i-1\right)}{\left(u_{k}^{\left(i,r-1\right)}\right)}^{2}{\left(B^{\left(i-1\right)}\right)}^{⊤}\right)+\epsilon^{2}I\right)}^{\frac{2-p}{2}}$.

**26**   Estimate $x_{k}^{\left(i,r\right)},\;P_{k|k}^{\left(i,r\right)}$ and $P_{k|k-1}^{\left(i,r\right)}$ using FIS.

**27**   Set $u_{k}^{\left(i,r\right)}=\text{max}\left(u_{\text{th}},{\left({B^{\left(i-1\right)}}^{⊤}B^{\left(i-1\right)}\right)}^{-1}{B^{\left(i-1\right)}}^{⊤}\left(x_{k}^{\left(i,r\right)}-Ax_{k-1}^{\left(i,r\right)}\right)\right)$.

**28**  **end**

**29**  **M-Step**: Set $x_{k}^{\left(i\right)}=x_{k}^{\left(i,r\right)},\;u_{k}^{\left(i\right)}=u_{k}^{\left(i,r\right)},\;P_{k|k}^{\left(i\right)}=P_{k|k}^{\left(i,r\right)}$ and $P_{k|k-1}^{\left(i\right)}=P_{k|k-1}^{\left(i,r\right)}$ and solve the optimization problem in Eq 17 to obtain obtain ${\tilde{\theta }}^{\left(i\right)}$.

**30 end**

#### Consideration of non-convexity

The complete data log-likelihood that is optimized by the EM approach might suffer from non-convexity and there is a potential risk that the solution may end up in different locations for different initial values. To test that, we run our EM approach for multiple random initializations of the physiological system parameters. Based on the simulated and the experimental datasets we have analyzed, we have observed that the solution for a given SC signal always converges to one location no matter what initial value has been selected. Therefore, we decided to only run our approach for one random initialization of the physiological system parameters in this study, unlike our previous approaches where we have used multiple random initializations and selected the solution that satisfies the selection criteria [23, 24, 28].

## Results

**Fig 2:**
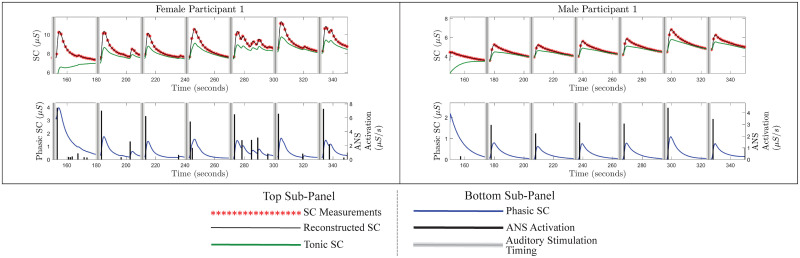
Estimated decomposition of the experimental SC signals for female participant 1 and male participant 1. In each of the panels, i) the top sub-panel shows the experimental SC signal (red stars), the reconstructed SC signal (black curve), the estimated tonic component (green curve), and the timings of the auditory stimulations (gray vertical lines); ii) the bottom sub-panel shows the estimated phasic component (blue curve), estimated ANS activation timings and amplitudes (black vertical lines) and the timings of the auditory stimuli (gray vertical lines).

We use the proposed approach to deconvolve the SC measurements from 26 participants. The deconvolution approach provides the estimates of the underlying ANS activation *u*(*t*), rise time (*τ*_*r*_), faster decay time (*τ*_*p*_), and slow decay time (*τ*_*d*_). We have considered the signal segment from 150 to 350 seconds for the analysis on the experimental data. Figures from the deconvolution results for one female and one male participant are provided in Fig 2. The figures from the deconvolution results for all 13 female and 13 male participants are provided in S1–S4 Figs. These figures depict the successful estimation of the sparse ANS activation due to auditory stimulation.

**Fig 3:**
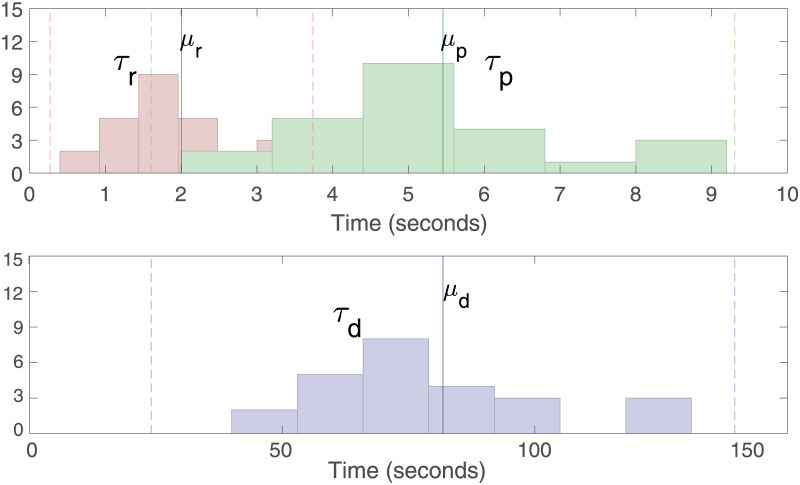
Histograms of estimated SCR shape parameters using our approach. In top sub-panel in the red and green bar plots correspond to the histogram plots of the estimated rise time *τ*_*r*_ and decay time *τ*_*d*_, respectively. Red, green and blue vertical lines correspond to the locations of the means *μ*_*r*_, *μ*_*p*_ and *μ*_*d*_ of the corresponding histograms, respectively.

The estimated rise time (*τ*_*r*_), fast decay time *τ*_*p*_, slow decay time *τ*_*d*_, number of pulses (||**u**||_0_), and multiple correlation coefficient (*R*^2^) are provided in Table 1. Fig 3 shows the histogram of the estimated state-space model parameters from all 26 participants. The estimated means of the parameters among the 26 participants are *μ*_*r*_ = 2.0040, *μ*_*p*_ = 5.4545, and *μ*_*d*_ = 81.8175 seconds for rise times, fast decay time, and slow decay times, respectively. Corresponding standard deviations are *σ*_*r*_ = 0.8675, *σ*_*p*_ = 1.9258, and *σ*_*d*_ = 28.8874 seconds, respectively. The calculated multiple correlation coefficients (*R*^2^) are greater than 0.98 for all participants except for Male Participant 12 (*R*^2^ for Male Participant 12 is 0.8352). This suggests that the proposed model can successfully explain the variations in SC recording.

**Table 1:** The estimated model parameters and the squares of the multiple correlation coefficients (*R*^2^) for the fits of the experimental SC data.

Female Participant	ID	*τ*_*r*_	*τ*_*p*_	*τ*_*d*_	||***u***||_0_	*R* ^2^
1	12	2.4575	6.4373	96.5591	25	0.9980
2	15	2.6889	6.9542	104.3135	24	0.9936
3	7	1.9565	5.3131	79.6968	28	0.9961
4	18	2.2324	5.9467	89.2004	25	0.9944
5	21	2.2948	6.0929	91.394	24	0.9893
6	25	2.3572	6.2167	93.2508	39	0.9990
7	1	1.3424	3.9588	59.3823	6	0.9986
8	2	0.7779	2.9288	43.9323	1	0.9883
9	5	1.2355	3.7123	55.6841	16	1
10	6	1.3411	3.9759	59.6391	11	0.9997
11	14	1.2101	3.6983	55.4741	9	0.9991
12	16	3.4221	8.6496	129.7442	41	0.9871
13	19	1.5775	4.4758	67.1366	25	0.9928
Male Participant	ID	*τ*_*r*_	*τ*_*p*_	*τ*_*d*_	||***u***||_0_	*R* ^2^
1	11	1.7215	4.7976	71.9641	8	0.9991
2	26	1.6574	4.6498	69.7463	13	0.9991
3	8	2.0524	5.5199	82.7989	24	0.9987
4	10	1.9070	5.2164	78.2453	40	0.9836
5	20	4.5170	11.0786	166.1788	59	0.9909
6	23	1.5451	4.4054	66.0803	27	0.9998
7	3	3.4100	8.6018	129.0276	58	0.9986
8	4	0.8936	3.1084	46.6253	8	0.9993
9	9	1.3561	4.0062	60.0935	20	0.9963
10	13	3.1618	8.066	120.9899	75	0.9954
11	17	1.6731	4.6962	70.4425	30	0.9976
12	22	1.7625	4.8939	73.4078	16	0.8352
13	24	1.5518	4.4164	66.2467	29	0.9992

Here *τ*_*r*_, *τ*_*p*_ and *τ*_*d*_, ||***u***||_0_, and *R*^2^ denote the rise time, fast decay time, slow decay time, ANS activation, and multiple correlation coefficients, respectively.

**Fig 4:**
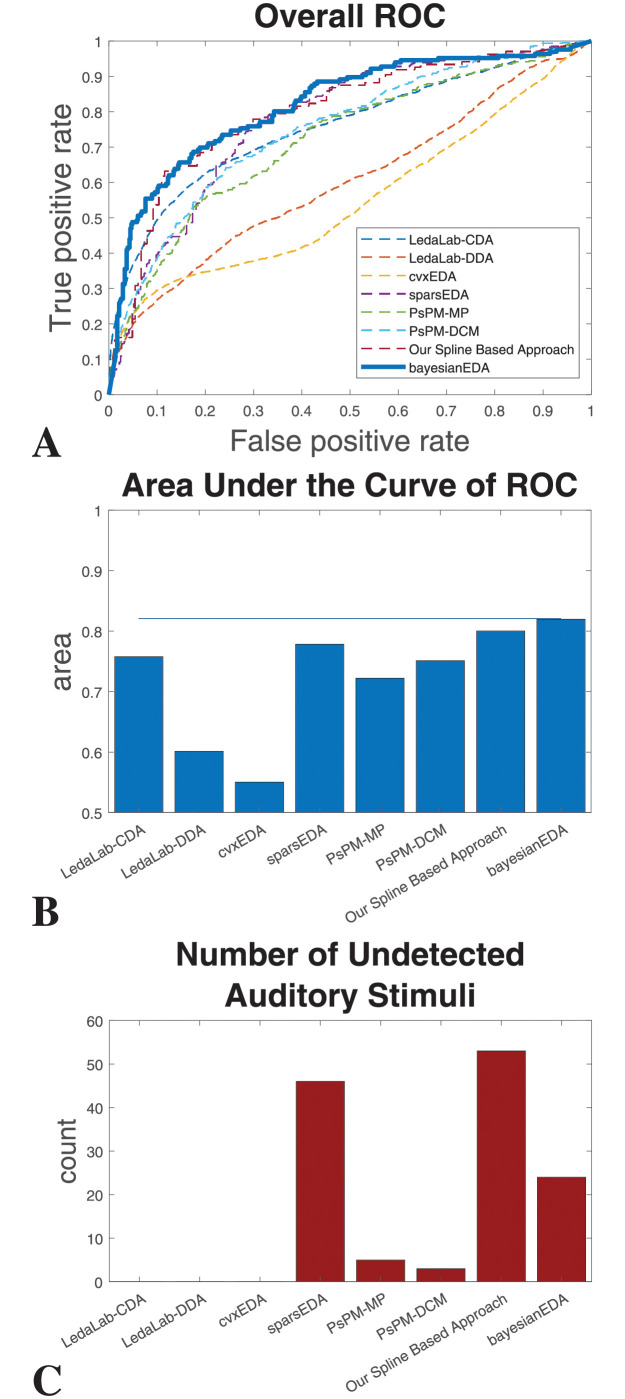
Event related SCR detection performance comparison. (A) The overall ROC curve related to the discrimination power between event-related vs non-event-related SCRs combining all the normalized **u** from each of the individual participants. (B) Corresponding AUC of the ROC curves. (C) Total number of the undetected auditory stimulation impulses within 26 participants.

For further evaluating the performance of the proposed algorithm on experimental data, we utilize it’s ability of separating a high-arousal condition (with larger ANS activation amplitudes) from a low-arousal condition (with smaller ANS activation amplitudes), inspired by the work commonly done in the PsPM framework [42]. We utilize the estimated ANS activation ***u***(*t*) in distinguishing between SCRs that are related to and not related to loud sound events. We label all the impulses in estimated ***u*** that have been detected within 5 seconds after a loud sound event as the positive class and other impulses as the negative class. We consider the amplitudes of the impulses as the classification scores within the subjects for obtaining the receiver operating characteristic (ROC) curves [43, 44]. The estimated area under the ROC curves (AUC) for all participants ranges from 0.6600 to 1 with a median of 0.9380 and a mean of 0.8960. We individually normalized the estimated ***u*** for all participant and combined all ***u*** in one vector to obtain an overall ROC. The estimated overall AUC is 0.8196. We compare our proposed bayesianEDA approach with LedaLab-CDA [19], LedaLab-DDA [26], cvxEDA [21], sparsEDA [34], PsPM-MP [45], PsPM-DCM [20], and our spline based approach [24]. The ROC curves are for each of the approaches shown in Fig 4A. The corresponding overall AUC’s are shown in Fig 4B. We further count the number of auditory stimulations for which no SCRs were detected, we name them as number of undetected auditory stimulation. The number of undetected auditory stimulation for each approaches is shown in Fig 4C.

**Fig 5:**
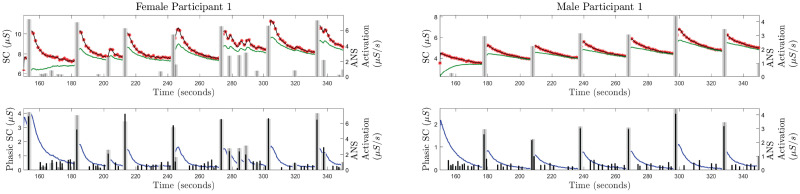
Deconvolution results from the simulated SC signals with 25 dB SNR for one female participant and one male participant. In each of the panels, i) the top sub-panel shows the ground truth for SC signal (red stars), the reconstructed SC signal (black solid curve), the estimated tonic component (green solid curve), and ground truth for the ANS activation (gray vertical lines); ii) the bottom sub-panel shows the estimated phasic component (blue solid curve), estimated ANS activation timings and amplitudes (black vertical lines) and the ground truth ANS activation (gray vertical lines).

**Fig 6:**
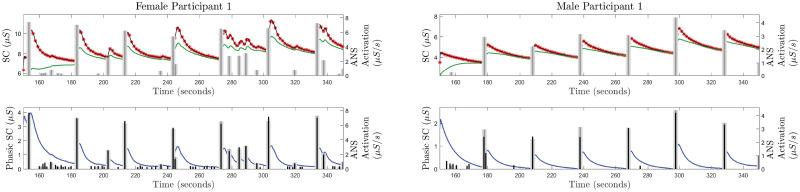
Deconvolution results from the simulated SC signals with 35 dB SNR for one female participant and one male participant. In each of the panels, i) the top sub-panel shows the ground truth for SC signal (red stars), the reconstructed SC signal (black solid curve), the estimated tonic component (green solid curve), and ground truth for the ANS activation (gray vertical lines); ii) the bottom sub-panel shows the estimated phasic component (blue solid curve), estimated ANS activation timings and amplitudes (black vertical lines) and the ground truth ANS activation (gray vertical lines).

To further investigate the efficacy of our approach, we use the reconstructed signal from our experimental study and add Gaussian noise to simulate data for all 26 participants similar to the previous works in [23, 24, 28, 46, 47]. We consider the results from the experimental study as the ground truths to compare with the estimation from the simulated study. The proposed approach successfully estimates the ANS activation along with the physiological model parameters. All the multiple correlation coefficients (*R*^2^) are greater than 0.98 for simulated data with 25 dB noise level is 0.9872. Estimated system parameters (${\hat{\tau }}_{r}$, ${\hat{\tau }}_{p}$ and ${\hat{\tau }}_{d}$), estimation errors, and the multiple correlation coefficients (*R*^2^) for the results for all the simulated data with 25 dB SNR are provided in Table 2. Further, we also perform the same analysis for 35 dB SNR noise level. The deconvolution result figures related to both 25 dB and 35 dB SNR noise level are also provided in Figs 5 and 6 for two participants for each case. All the other simulation results with Gaussian noise are provided in S5–S12 Figs. Furthermore, we performed similar deconvolution study with pink noise with 25 dB SNR for the signal which show similar results as for the case of Gaussian noise showing the robustness to the model mismatch. The corresponding figures are provided in S13–S16 Figs.

**Table 2:** The estimated model parameters, estimation errors, and the squares of the multiple correlation coefficients (*R*^2^) for the fits of the simulated SC data.

Female Participant	ID	*τ*_*r*_	*τ*_*p*_	*τ*_*d*_	$\frac{|\tau_{r}-{\hat{\tau }}_{r}|}{\tau_{r}}\times 100\%$	$\frac{|\tau_{p}-{\hat{\tau }}_{p}|}{\tau_{p}}\times 100\%$	$\frac{|\tau_{d}-{\hat{\tau }}_{d}|}{\tau_{d}}\times 100\%$	*R* ^2^	run time
1	12	2.4604	6.4389	96.5830	0.1210	0.0247	0.0247	0.99794	31.6575
2	15	2.6990	6.9523	104.2847	0.3778	0.0276	0.0276	0.99755	317
3	7	1.9586	5.3138	79.7069	0.1071	0.0126	0.0126	0.99755	29.5756
4	18	2.2347	5.9467	89.2011	0.1044	0.0008	0.0008	0.99688	30.5766
5	21	2.3006	6.0931	91.3963	0.2512	0.0025	0.0025	0.99363	28.2613
6	25	2.3588	6.2170	93.2545	0.0693	0.0040	0.0040	0.99789	30.4497
7	1	1.3436	3.9595	59.3931	0.0900	0.0182	0.0182	0.99970	26.1312
8	2	0.7779	2.9288	43.9316	0.0018	0.0016	0.0016	0.99830	21.3974
9	5	1.2366	3.7137	55.7056	0.0907	0.0388	0.0388	0.99888	21.5944
10	6	1.3411	3.9762	59.6431	0.0036	0.0067	0.0067	0.99985	24.9445
11	14	1.2102	3.6981	55.4716	0.0079	0.0045	0.0044	0.99976	28.5442
12	16	3.4366	8.6424	129.6358	0.4253	0.0836	0.0836	0.98704	34.1906
13	19	1.5792	4.4764	67.1456	0.1075	0.0133	0.0133	0.99814	26.3280
Male Participant	ID	*τ*_*r*_	*τ*_*p*_	*τ*_*d*_	$\frac{|\tau_{r}-{\hat{\tau }}_{r}|}{\tau_{r}}\times 100\%$	$\frac{|\tau_{p}-{\hat{\tau }}_{p}|}{\tau_{p}}\times 100\%$	$\frac{|\tau_{d}-{\hat{\tau }}_{d}|}{\tau_{d}}\times 100\%$	*R* ^2^	run time
1	11	1.7232	4.7982	71.9732	0.0995	0.0126	0.0126	0.99906	25.9000
2	26	1.6579	4.6494	69.7406	0.0312	0.0082	0.0082	0.99911	29.7408
3	8	2.0574	5.5219	82.8286	0.2476	0.0358	0.0358	0.99864	30.1971
4	10	1.9103	5.2170	78.2550	0.1727	0.0125	0.0125	0.98358	28.9428
5	20	4.5459	11.0648	165.9723	0.6384	0.1242	0.1242	0.99098	32.0364
6	23	1.5452	4.4053	66.0799	0.0042	0.0006	0.0006	0.99983	29.6168
7	3	3.4207	8.5952	128.9286	0.3125	0.0767	0.0767	0.99864	32.2376
8	4	0.8937	3.1084	46.6255	0.0026	0.0004	0.0004	0.99938	23.1102
9	9	1.3568	4.0064	60.0960	0.0571	0.0042	0.0042	0.99632	26.4126
10	13	3.1736	8.0676	121.0135	0.3727	0.0195	0.0195	0.99549	31.7452
11	17	1.6754	4.6976	70.4639	0.1386	0.0304	0.0304	0.99762	26.2041
12	22	1.7660	4.8959	73.4382	0.1990	0.0414	0.0414	0.83526	24.6898
13	24	1.5524	4.4157	66.2352	0.0409	0.0174	0.0174	0.99922	28.8487

Here ${\hat{\tau }}_{r}$, ${\hat{\tau }}_{p}$ and ${\hat{\tau }}_{d}$ denote the estimated rise time, fast decay time, and slow decay time for the simulated SC data. The SC signal is simulated with 25 dB Gaussian noise.

**Fig 7:**
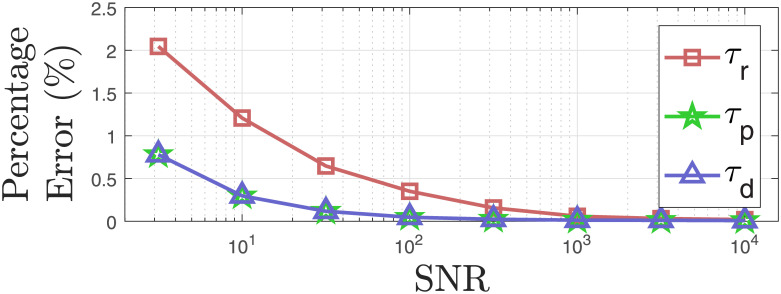
Noise levels vs. estimation accuracy of the model parameters. Red squares, green pentagram, and blue triangles connected with solid lines denote the average percentage errors for the estimated rise times, fast decay times, and slow decay time from simulated data with SNR levels. The SNR is provided with respect to the phasic component.

**Fig 8:**
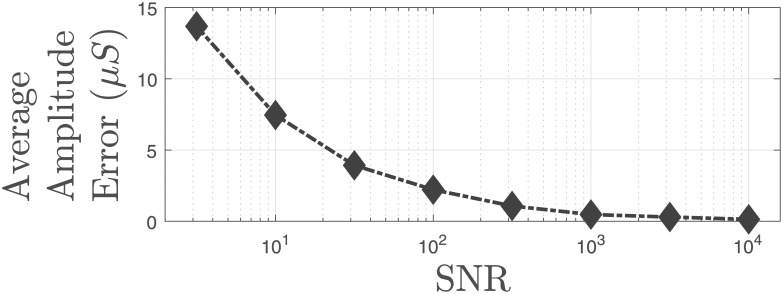
Average amplitude error of estimated ANS activation in different noise levels. Black diamonds with the dashed lines denotes the average amplitude error of the neural stimuli from estimated data with different noise levels. We have defined the average amplitude error as $|{\left\|\tilde{u}\right\|}_{1}-{\left\|u\right\|}_{1}|/{\left\|u\right\|}_{0}$, where $\tilde{u}$ and ***u*** represent the estimated and the ground truth neural stimuli, respectively. The data is simulated using the obtained results from the all experimental data in [31]. The SNR is given with respect to the phasic component.

**Fig 9:**
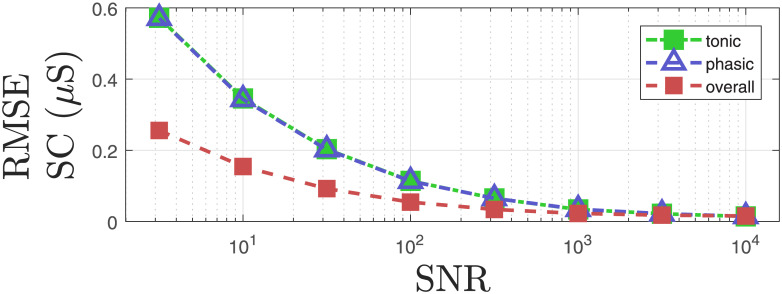
Root mean square error (RMSE) of the reconstruction for SC signal and corresponding components with respect to the ground truth. Green, blue and red dashed lines denote the RMSE for the reconstructed tonic component, phasic component and overall SC data in different noise levels. The data is simulated using the obtained results from the all experimental data in [31]. As noise is added to the phasic component prior to addition of tonic component, the SNR is given with respect to the phasic component.

We add noise with different noise power to investigate how the proposed approach performs in terms of estimating the unknowns and the reconstructed signal. We add Gaussian noise with different energy levels to the reconstructed SC signals from the experimental study for the 26 participants and perform deconvolution to estimate unknowns with the proposed approach. We calculate the average estimation errors of the unknowns for all participants at different noise levels. Figs 7 and 8 show how the average estimation error changes as the noise level increases. Similarly, Fig 9 shows how the reconstruction errors change at different noise levels.

**Fig 10:**
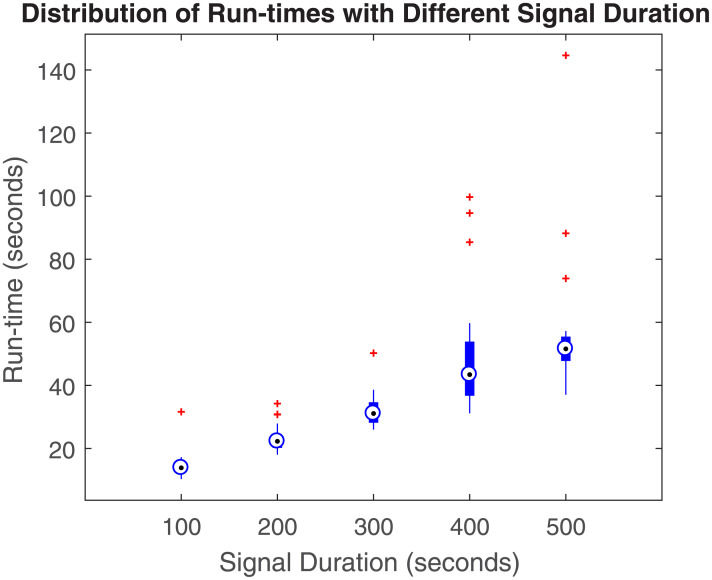
Run-time vs signal length. Figure shows boxplots of the run-times of the proposed approach with different signal lengths. The black dots with blue circle in the middle of each boxplot denote median. The bottom and top of each blue box are the 25th and 75th percentiles of the sample, respectively. The red markers denote the outliers.

To empirically investigate the time complexity of the approach, we utilize the experimental data with different durations and perform deconvolution using our approach. We measure the run-time for each of the deconvolution. Fig 10 shows the distributions of the run-times in different signal lengths. According to the Fig 10, the medians of the run-times increase linearly with the increase in the signal length showing the scalability of the approach. For the signal with 200 second length, the mean run-time for M-step (parameter estimation step) is 0.38 seconds with a standard deviation of 0.15 seconds.

## Discussion

Inference of ANS activation from SC recordings is challenging given that the parameters of the underlying physiological system are unknown. The derived EM approach maximizes the complete data log-likelihood. The complete data log-likelihood has many degrees of freedom, i.e., the constraints on variables to be optimized are lower than the number of variables. In other words, there exist many solutions for the unknowns that can closely approximate the sampled signal. The use of a comprehensive state-space model and the elimination of cubic spline functions-based model reduces the number of unknown variables in optimization. For example, the number of cubic spline functions needed to model the slow varying component of 200 seconds is 39, as pointed out in our previous work [24]. On the other hand, the proposed comprehensive model requires only one parameter instead of multiple cubic spline function parameters to model the slow-varying component. Furthermore, we consider probabilistic sparsity priors motivated by physiology on ANS activation along with Gaussian priors on the physiological system parameters. Last but not least, we also enforce inequality and equality constraints on the state-space model parameters by trial and error. The constraints *τ*_*p*_ > 2*τ*_*r*_, *τ*_*d*_ > 15*τ*_*p*_, and *η* = 0.5 worked best for us for the dataset we have analyzed. [24]. [24].

Fig 2 shows that the estimations of the initial states as well as the states for about 20–30 seconds can be erroneous. After 20–30 seconds, the state estimate visually seems reasonable. This erroneous estimation occurs because the Kalman filter in the FIS needs a few samples to begin to follow the signal. Therefore, the estimations during the initial few samples can be erroneous. Due to this erroneous estimation of the initial state, the *R*^2^ estimate for male participant 12 became very low compared to other participants. One straightforward way to deal with this is to consider 20–30 seconds of measured signal padded in the beginning. After performing deconvolution in the padded signal, results corresponding to the initial 20–30 seconds can be removed.

For the comparative study with previous approaches, we assumed the timing of the auditory stimulation as the ground truth. It should be noted that the shape of the ROC curve is dictated by the three factors: 1) how many of the auditory stimuli are translated as SCRs by the neural pathway and corresponding physiology, 2) spontaneous SCRs, and 3) an algorithm’s ability to accurately model any SCRs along with the corresponding accurate estimation of ANS activations. If an auditory stimulation does not produce an SCR, all different algorithms will be penalized the same way in the ROC metric if that specific SCR is not detected (contributing as the false negative). Similarly, if there is a spontaneous SCR, all different algorithms will be penalized the same way in the ROC metric if detected (contributing as the false positive). As the first two cases are staying the same for all the algorithms, the relative change in the area in the AUC of the ROC curve will mean that this change is coming from the algorithm itself only. In this way we can benchmark our approach with previous algorithms. A better ROC will mean algorithms ability to reduce the false negatives and false positives. Fig 4A shows that our bayesianEDA has the best ROC curve than all the previous approaches, including our previously proposed spline-based approach [24]. Fig 4B shows that our bayesianEDA has the maximum AUC value of the corresponding ROC curves. The next best ones are our spline-based approach (AUC = 0.8003) and sparsEDA (AUC = 0.7783). The ROC curves and AUC values are generated based only on the classification ability between the event-related and non-event related SCRs among the ones that are only detected by each method. However, there is a possibility that an algorithm have over-sparsified the solution and missed many smaller but event-related SCRs. Therefore, we further calculate for how many of auditory stimulations no SCR was detected. Fig 4C shows that among all algorithms, our BayesianEDA approach has 24 undetected ANS activation, which is close to the correct number of undetected responses, which is 23. Detailed discussion is provided in S3 Appendix.

Readers should note that, unlike all the methods we considered for the comparison, PsPM [20, 45] was specifically developed to incorporate knowledge of external stimulation, and the dataset used comes from an experiment with defined stimulation. PsPM can utilize this defined stimulation information. All other approaches including ours perform “blind” deconvolution regardless of any external stimulation. This is more applicable in the envisioned application area, such as real-time deconvolution with wearables. It can also be thought of as a drawback when there is knowledge of stimulation, such as in most laboratory tasks. Therefore, here we used the spontaneous fluctuation (SF) suite for PsPM for our comparison, which also does not take the information of external stimulus as input. In the future, inspired by the PsPM framework, we plan to extend our proposed algorithm bayesianEDA to take the stimulus information as input in a probabilistic manner by changing the probability distribution of *u*(*t*) at the time when external stimulation information exists for a more contex-aware deconvolution.

The computational complexity of the deconvolution approach is O(K) as shown in [30]. Furthermore, our empirical investigation also shows that the run-time scales linearly with the number of samples, as shown in Fig 10. This shows the feasibility of implementing such approaches in low-power wearable medical devices for edge computation. This scalable implementation has been possible with the proposed comprehensive state-space model. The time complexity of the M-step of this approach is also of O(K) in terms of the number of samples. After E-step the calculation of the summations such as $\sum_{k=0}^{K-1}\left(x_{k-1}^{\left(i\right)}{\left(x_{k-1}^{\left(i\right)}\right)}^{⊤}+P_{k-1}^{\left(i\right)}\right)$, $\sum_{k=0}^{K-1}y_{k}^{⊤}Cx_{k}^{\left(i\right)}$ etc. in Eq 17 has O(K) time complexity. Further optimization can be performed by obtaining the parameters of the physiological system for a smaller segment and performing the E-step for the longer segments. During a day of recording, parameters can be updated a few times by running the EM, and these parameters can be used to estimate the ANS activation using only E-step. A real-time implementation can be done with only running the Kalman filter in an iterative manner in the FIS after estimating the system parameters for a shorter segment. As Kalman filters are very cheap in terms of computation power, the proposed approach opens up the possibility of performing ANS activity inference on the edge device rather than running it in the cloud, facilitating low network traffic and user privacy.

In this study, we have proposed a novel physiological model inspired by the physiological understanding of sweat secretion that can better explain the variation in SC with fewer unknowns. Using our proposed model, we have developed a highly scalable deconvolution algorithm, which will enable efficient implementation in wearable devices. To achieve convergence, obtain a good fit of the model, and avoid overfitting, several parameters and constraint have been chosen on a trial-and-error basis because of the absence of in-depth physiological knowledge. There is room for improvement to come up with a more systematic way to address this limitation. Future studies can benefit from more motivation from physiology-motivated parameters and constraint selections.

ANS activities obtained from the single channel SC recording can be used to track the cognitive arousal state of an individual [2, 48, 49]. One of the future goals is to extend this approach for multi-channel SC recording and the nonlinearity of the model for a more robust inference in the presence of noise,leading to more reliable inference of individual arousal level similar to our previous study in [28]. For further accurate estimate of emotional arousal, we intend to utilize the inferred ANS activity from SC recordings with our approach and combine with other physiological signals similar to [50–56]. The proposed new model as well as the scalable ANS inference approach have enabled us to design a scalable control architecture to regulate the arousal level similar to the proposed framework in [57–60]. Finally, since some studies have reported inconsistencies in the *poral valve model* by Edlberg et al. [33] while investigating both SC and skin potential response [61], we plan to continue our investigation of the mechanism of sweat secretion to achieve improvements in the model and its understanding.

## Supporting information

S1 Appendix Expectation maximization. The section provides a brief derivation of the Expectation Maximization.
(PDF)

S2 Appendix Heuristic refinement of *u*. The section provide a brief derivation of the heuristic refinement of ***u***.
(PDF)

S3 Appendix Additional discussion. The section provides a detailed discussion on performance comparison between different algorithms in terms of the number of undetected activations of ANS.
(PDF)

S1 Fig Estimated decomposition of the experimental SC signals for female participant 1 to 6. (PDF)

S2 Fig Estimated decomposition of the experimental SC signals for female participant 7 to 13. (PDF)

S3 Fig Estimated decomposition of the experimental SC signals for male participant 1 to 6. (PDF)

S4 Fig Estimated decomposition of the experimental SC signals for male participant 7 to 13. (PDF)

S5 Fig Deconvolution results from the simulated SC signals with 25 dB SNR for female participant 1 to 6. (PDF)

S6 Fig Deconvolution results from the simulated SC signals with 25 dB SNR for female participant 7 to 13. (PDF)

S7 Fig Deconvolution results from the simulated SC signals with 25 dB SNR for male participant 1 to 6. (PDF)

S8 Fig Deconvolution results from the simulated SC signals with 25 dB SNR for male participant 7 to 13. (PDF)

S9 Fig Deconvolution results from the simulated SC signals with 35 dB SNR for female participant 1 to 6. (PDF)

S10 Fig Deconvolution results from the simulated SC signals with 35 dB SNR for female participant 7 to 13. (PDF)

S11 Fig Deconvolution results from the simulated SC signals with 35 dB SNR for male participant 1 to 6. (PDF)

S12 Fig Deconvolution results from the simulated SC signals with 35 dB SNR for male participant 7 to 13. (PDF)

S13 Fig Deconvolution results from the simulated SC signals with 25 dB SNR with pink noise for female participant 1 to 6. (PDF)

S14 Fig Deconvolution results from the simulated SC signals with 25 dB SNR with pink noise for female participant 7 to 13. (PDF)

S15 Fig Deconvolution results from the simulated SC signals with 25 dB SNR with pink noise for male participant 1 to 6. (PDF)

S16 Fig Deconvolution results from the simulated SC signals with 25 dB SNR with pink noise for male participant 7 to 13. (PDF)
