# Supplementary material for: Physiological characterization of electrodermal activity enables scalable near real-time autonomic nervous system activation inference
Source: PLoS Comput Biol. 2022 Jul 28;18(7):e1010275. doi: 10.1371/journal.pcbi.1010275 (PMC9333288; doi:10.1371/journal.pcbi.1010275)
Supplement: S1 Appendix — The section provides a brief derivation of the Expectation Maximization. (PDF) [file pcbi.1010275.s001.pdf]

Supplementary Information - Physiological Characterization of Electrodermal Activity Enables Scalable Near Real-Time Autonomic Nervous System Activation Inference

Md. Rafiul Amin[1], Rose T. Faghih[1‡*],

1 Department of Electrical and Computer Engineering, University of Houston, Houston, Texas, USA

S1 Appendix. Expectation Maximization. We can re-write the maximum likelihood estimation as the following marginal likelihood function of $p(Y, X; \theta)$:

$$\max_{\theta} \log p(Y; \theta) = \max_{\theta} \log \int_X p(Y, X; \theta) dX. \tag{1}$$

Obtaining the marginal likelihood by the integration operation in Equation 1 is difficult, especially with edge computation on wearable devices or smart-phones. This problem is usually modified as follows:

$$\begin{aligned} &\max_{\theta} \log \int_X p(Y, X; \theta) dX = \max_{\theta} \log \left(\int_X \frac{p(Y, X; \theta)}{q(X)} q(X) dX \right) \\ &\geq \max_{\theta} \int_X q(X) \log \left(\frac{p(Y, X; \theta)}{q(X)} \right) dX \quad \text{[Jensen's inequality]} \\ &= \underbrace{\max_{\theta} \int_X q(X) \log \left(p(Y, X; \theta) \right) dX}_{\text{function of } \theta} - \underbrace{\int_X q(X) \log \left(q(X) \right) dX}_{\text{constant}}, \end{aligned}$$

where $q(X)$ is any probability density function. Therefore, the original problem is defined as the following expectation maximization (EM) approach,

$$\max_{\theta} \log p(Y; \theta) = \max_{\theta} \mathbb{E}_{X \sim q(X)} \{ \log p(Y, X; \theta) \}. \tag{2}$$

As it is expressed in Equation 2, the unknowns can be estimated by iteratively maximizing the expectation of the joint log-likelihood $\log p(Y, X; \theta)$.
