# Supplementary material for: Physiological characterization of electrodermal activity enables scalable near real-time autonomic nervous system activation inference
Source: PLoS Comput Biol. 2022 Jul 28;18(7):e1010275. doi: 10.1371/journal.pcbi.1010275 (PMC9333288; doi:10.1371/journal.pcbi.1010275)
Supplement: S2 Appendix — The section provide a brief derivation of the heuristic refinement of u. (PDF) [file pcbi.1010275.s002.pdf]

Supplementary Information - Physiological Characterization of Electrodermal Activity Enables Scalable Near Real-Time Autonomic Nervous System Activation Inference

Md. Rafiul Amin[1], Rose T. Faghih[1‡*],

1 Department of Electrical and Computer Engineering, University of Houston, Houston, Texas, USA

S2 Appendix. Heuristic Refinement of *u*. Similar to our previous deconvolution approaches [1–3], we perform a heuristic refinement to enforce a constraint on the maximum number of nonzero values in $\boldsymbol{u}$ and a minimum spacing between two consecutive impulses. In each iteration of the re-weighting step, we perform the following two steps for enforcing these constraints:

- Detect the nonzero values having time distance less than the selected minimum peak to peak distance Δ_p ($\Delta_p = 1$ second in our case). Retain only the largest impulses among the adjacent impulses within the Δ_p window.
- If $||\mathbf{u}^{(i,r)}||_0 > N_{\mathbf{u}}^{\max}$, select $N_{\mathbf{u}}^{\max}$ largest values of elements of $\mathbf{u}^{(i,r)}$ and set all other elements to zero.

We choose $N_{\mathbf{u}}^{\max} = N_{\text{peaks}} + 20$. N_{peaks} is the number of peaks detected on the raw SC recording using MATLAB *findpeaks* function with a peak prominance of 5×10^{-4} and a peak to peak distance of 1 second. Further, we have selected $u_{th} = 0.03$ in this study for thresholding.

References

1. Amin MR, Faghih RT. Identification of Sympathetic Nervous System Activation from Skin Conductance: A Sparse Decomposition Approach with Physiological Priors. IEEE Transactions on Biomedical Engineering. 2020;.
2. Amin MR, Faghih RT. Robust Inference of Autonomic Nervous System Activation Using Skin Conductance Measurements: A Multi-Channel Sparse System Identification Approach. IEEE Access. 2019;7:173419–173437.
3. Amin MR, Faghih RT. Sparse Deconvolution of Electrodermal Activity via Continuous-Time System Identification. IEEE Transactions on Biomedical Engineering. 2019;.
