# Supplementary material for: Physiological characterization of electrodermal activity enables scalable near real-time autonomic nervous system activation inference
Source: PLoS Comput Biol. 2022 Jul 28;18(7):e1010275. doi: 10.1371/journal.pcbi.1010275 (PMC9333288; doi:10.1371/journal.pcbi.1010275)
Supplement: S3 Appendix — The section provides a detailed discussion on performance comparison between different algorithms in terms of the number of undetected activations of ANS. (PDF) [file pcbi.1010275.s003.pdf]

Supplementary Information - Physiological Characterization of Electrodermal Activity Enables Scalable Near Real-Time Autonomic Nervous System Activation Inference

Md. Rafiul Amin[1◑], Rose T. Faghih[1‡*],

1 Department of Electrical and Computer Engineering, University of Houston, Houston, Texas, USA

S3 Appendix Additional Discussion Figure 4-C in main article shows, our spline previous approach is missing most of the auditory stimulation (total of 53) and sparsEDA [1] misses the second most number of auditory stimulation (total of 46). On the other hand, LedaLab-CDA [2], LedaLab-DDA [3], and cvxEDA [4] do not miss any of the auditory stimulation. Note that sometimes a participant might not show any SCR in response to an auditory stimulus. Therefore, a fraction of the number of undetected auditory stimulation can be attributed to the participant's physiology, and the rest can be attributed to the algorithm itself. This observation reveals that LedaLab-CDA, LedaLab-DDA, and cvxEDA detect noise spikes as SCRs in cases where participants do not have an SCR response. From our visual investigation, we see that some of the participants does not have any SCR in response to the auditory simulation. Please see the experimental results for Female participant 7, 8, 10, 11, 13 and Male participant 8 in Figure S2 and Figure S4. Our approach does not detect a total of 24 auditory stimuli and most of them can be attributed to no SCR response from the participant. On the other hand, sparsEDA and our spline-based approach have missed 46 and 53 numbers of stimuli, respectively, resulting in an overly sparse solution. By visual inspection, we observe that 23 of all auditory stimuli do not lead to an SCR, which is very close to the number of auditory stimuli not detected by our bayesianEDA.

References

1. Hernando-Gallego F, Luengo D, Artés-Rodríguez A. Feature Extraction of Galvanic Skin Responses by Non-Negative Sparse Deconvolution. IEEE Journal of Biomedical and Health Informatics. 2017;.
2. Benedek M, Kaernbach C. A continuous measure of phasic electrodermal activity. Journal of neuroscience methods. 2010;190(1):80–91.
3. Benedek M, Kaernbach C. Decomposition of skin conductance data by means of nonnegative deconvolution. Psychophysiology. 2010;47(4):647–658.
4. Greco A, Valenza G, Lanata A, Scilingo EP, Citi L. cvxEDA: A convex optimization approach to electrodermal activity processing. IEEE Transactions on Biomedical Engineering. 2016;63(4):797–804.
