# Supplementary material for: Physiological characterization of electrodermal activity enables scalable near real-time autonomic nervous system activation inference
Source: PLoS Comput Biol. 2022 Jul 28;18(7):e1010275. doi: 10.1371/journal.pcbi.1010275 (PMC9333288; doi:10.1371/journal.pcbi.1010275)
Supplement: S4 Fig — (PDF) [file pcbi.1010275.s007.pdf]

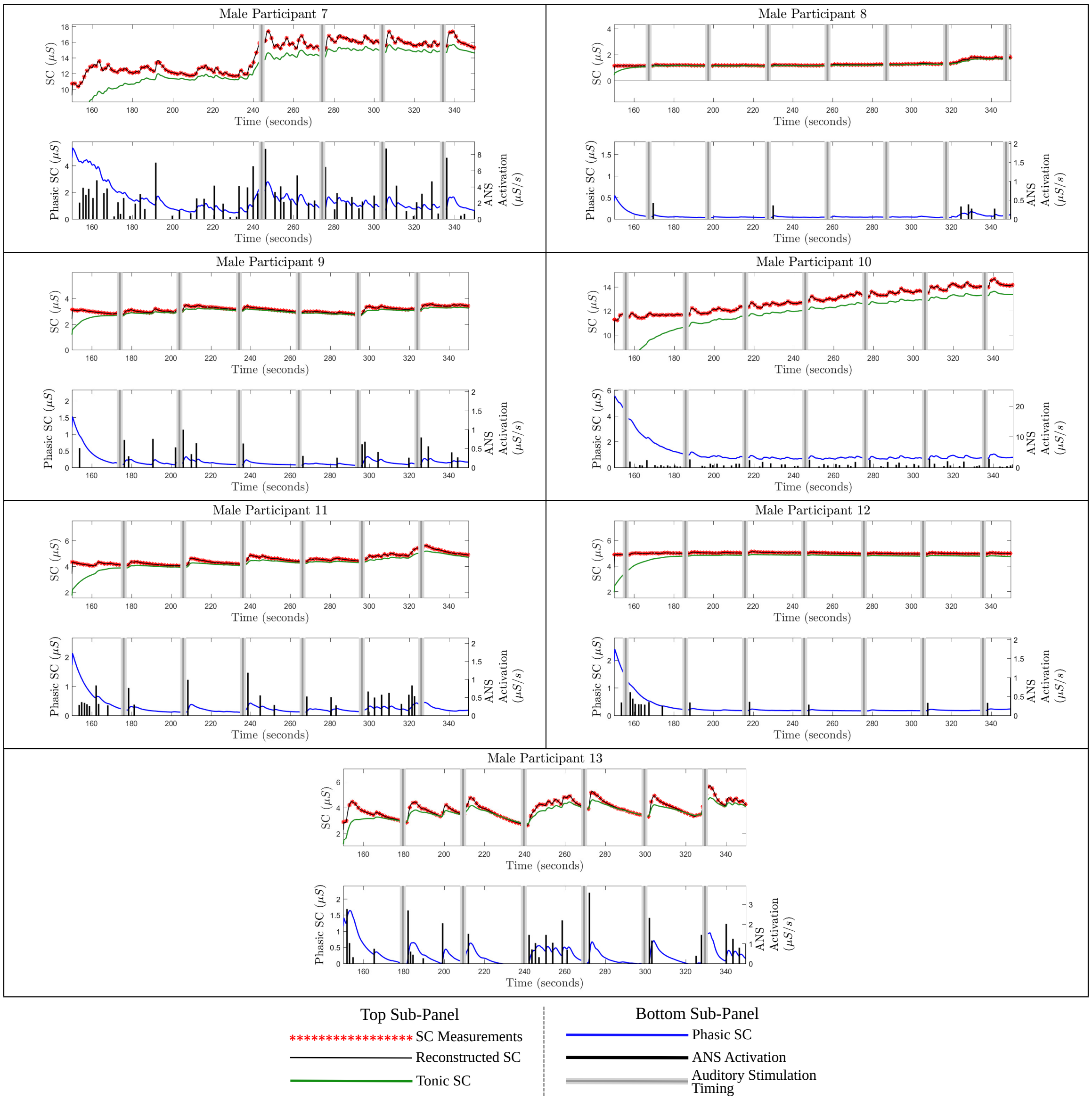


**Fig S4. Estimated Decomposition of the Experimental SC Signals for Male Participant 7 to 13:** In each of the panels, i) the top sub-panel shows the experimental SC signal (red stars), the reconstructed SC signal (black curve), the estimated tonic component (green curve), and the timings of the auditory stimulations (gray vertical lines); ii) the bottom sub-panel shows the estimated phasic component (blue curve), estimated ANS activation timings and amplitudes (black vertical lines) and the timings of the auditory stimuli (gray vertical lines).
