# Supplementary material for: Physiological characterization of electrodermal activity enables scalable near real-time autonomic nervous system activation inference
Source: PLoS Comput Biol. 2022 Jul 28;18(7):e1010275. doi: 10.1371/journal.pcbi.1010275 (PMC9333288; doi:10.1371/journal.pcbi.1010275)
Supplement: S16 Fig — (PDF) [file pcbi.1010275.s019.pdf]

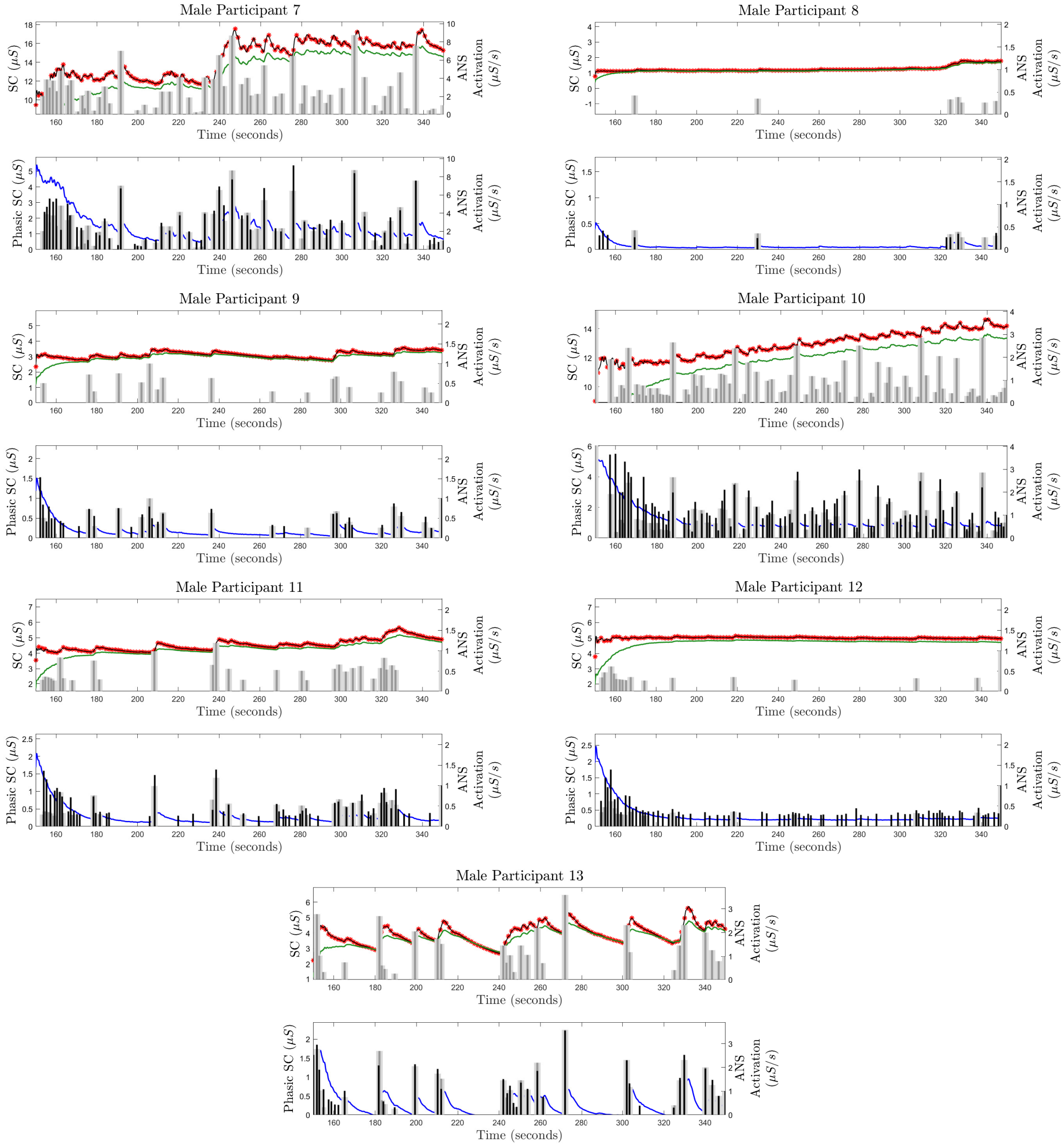

Fig S16. Deconvolution Results From the Simulated SC Signals with 25 dB SNR with Pink Noise for Male Participant 7 to 13: In each of the panels, i) the top sub-panel shows the ground truth for SC signal (red stars), the reconstructed SC signal (black solid curve), the estimated tonic component (green solid curve), and ground truth for the ANS activation (gray vertical lines); ii) the bottom sub-panel shows the estimated phasic component (blue solid curve), estimated ANS activation timings and amplitudes (black vertical lines) and the ground truth ANS activation (gray vertical lines).

.
